# Diffusion of hydrocarbons diluted in supercritical carbon dioxide

**DOI:** 10.1038/s41598-023-42892-7

**Published:** 2023-09-26

**Authors:** Denis Saric, Gabriela Guevara-Carrion, Yury Gaponenko, Valentina Shevtsova, Jadran Vrabec

**Affiliations:** 1 Thermodynamics, Technical University of Berlin, Ernst-Reuter-Platz 1, 10587 Berlin, Germany; 2 MRC, CP-165/62, Université libre de Bruxelles (ULB), Ave. F.D. Roosevelt 50, B-1050 Brussels, Belgium; 3 Fluid Mechanics Group, Faculty of Engineering, Mondragon University, 20500 Mondragon, Spain; 4 IKERBASQUE, Basque Foundation for Science, Plaza Euskadi 5, 48009 Bilbao, Spain

**Keywords:** Chemical physics, Thermodynamics

## Abstract

Mutual diffusion of six hydrocarbons (methane, ethane, isobutane, benzene, toluene or naphthalene) diluted in supercritical carbon dioxide (${\hbox {CO}}_{2}$) is studied by molecular dynamics simulation near the Widom line, i.e., in the temperature range from 290 to 345 K along the isobar 9 MPa. The ${\hbox {CO}}_{2}$ + aromatics mixtures are additionally sampled at 10 and 12 MPa and an experimental database with Fick diffusion coefficient data for those systems is provided. Taylor dispersion experiments of ${\hbox {CO}}_{2}$ with benzene, toluene, n-dodecane and 1,2,3,4-tetrahydronaphthalene are conducted along the $p =$ 10 MPa isobar. Maxwell–Stefan and Fick diffusion coefficients are analyzed, together with the thermodynamic factor that relates them. It is found that the peculiar behavior of the Fick diffusion coefficient of some ${\hbox {CO}}_{2}$ mixtures in the extended critical region is a consequence of the thermodynamic factor minimum due to pronounced clustering on the molecular scale. Further, the strong dependence of the Fick diffusion coefficient on the molecular mass of the solute as well as the breakdown of the Stokes–Einstein relation near the Widom line are confirmed. Eleven correlations for the prediction of the Fick diffusion coefficient of ${\hbox {CO}}_{2}$ mixtures are assessed. An alternative two-step approach for the prediction of the infinite dilution Fick diffusion coefficient of supercritical ${\hbox {CO}}_{2}$ mixtures is proposed. It requires only the state point in terms of temperature and pressure (or density) as well as the molecular solute mass as input parameters. First, entropy scaling is applied to estimate the self-diffusion coefficient of ${\hbox {CO}}_{2}$. Subsequently, this coefficient is used to determine the infinite dilution Fick diffusion coefficient of the mixture, based on the finding that these two diffusion coefficients exhibit a linear relationship, where the slope depends only on the molecular solute mass.

## Introduction

Carbon dioxide (${\hbox {CO}}_{2}$) has unique properties that make it an attractive working fluid for various supercritical fluid (SCF) applications^1^. It consists of small, linear molecules with enhanced diffusivity and has a relatively mild critical point ($T_{\text {c}}= 304.13$ K, $p_\text {c}= 7.38$ MPa, $\rho _\text {c}=$ 467.6 kg/m$^{3}$). Moreover, it is chemically stable, environmentally friendly and effectively absorbs many solute species. Hence, supercritical ${\hbox {CO}}_{2}$ (scCO_2_) can replace water in textile dyeing^2^ or serve as an alternative to organic solvents in separation processes^3^.

In practical industrial scenarios, ${\hbox {CO}}_{2}$ often contains impurities, such as traces of hydrocarbons, which can significantly affect the thermophysical behavior of ${\hbox {CO}}_{2}$ mixtures in their extended critical region. Understanding diffusion in ${\hbox {CO}}_{2}$ + hydrocarbon mixtures under supercritical conditions is thus crucial for the development, control and optimization of processes like enhanced oil recovery^4^ or ${\hbox {CO}}_{2}$ sequestration^5^. However, mutual diffusion coefficient data of such systems, particularly near the critical point, are scarce^6^. Experimental literature data are primarily limited to the low-temperature, high-density region, resulting in a lack of data for the low-density, high-temperature SCF region. Bridging this gap is essential for emerging SCF processes^7^.

Several semi-empirical correlations of the Stokes–Einstein^8–15^ or free-volume type^16–18^ have been developed to estimate the Fick diffusion coefficient of ${\hbox {CO}}_{2}$ mixtures. These correlations are simple, but do not rely on a molecular-scale understanding of the underlying diffusion process. Moreover, they often fail to accurately reproduce the Fick diffusion coefficient of sc${\hbox {CO}}_{2}$ mixtures^19,20^, especially near the critical point. For instance, the Stokes–Einstein-type correlations are mainly suited for the liquid-like region^7^, but tend to fail at low density^21^. Therefore, acquiring new data is necessary to critically reassess existing correlations and to develop improved ones^7^.

The lack of mutual diffusion coefficient data for high-pressure ${\hbox {CO}}_{2}$ + hydrocarbon mixtures is in stark contrast to their importance for reservoir processes^20^. The temperature, pressure and composition dependencies of the Fick diffusion coefficient remain largely unexplored over a wide range of thermodynamic conditions^22^. Experimental studies on ${\hbox {CO}}_{2}$ mixtures with benzene^12,18,23–32^, toluene^5,13,25,26, 32,33^ or naphthalene^12,18,34–41^ at high pressure (see Table  1) have provided valuable insights into the Fick diffusion coefficient, revealing a peculiar behavior in certain near-critical regions^28,30,42^, a significant dependence on the molecular mass of the solute^7,14,24,43^ and deviations from the conventional Stokes–Einstein behavior^7,22,31,44–46^.

In the extended critical region, a slight increase of temperature along a supercritical isobar may substantially reduce the density and shear viscosity, while it rises the intra-diffusion coefficients of the mixture’s components^47^. In that region, SCF can exhibit liquid-like (high density, high viscosity and low diffusivity) or gas-like (low density, low viscosity and high diffusivity) characteristics^47–51^. These regions are demarcated by the so-called Widom line, which can be considered as an extension of the vapor pressure curve up to approximately $10 \cdot p_c$^52^. Close to the Widom line pronounced density fluctuations occur^53–55^, leading to a strongly non-ideal behavior of the mixture^56,57^ and large changes of its thermodynamic and transport properties^47,58–60^. One well-known example is the isobaric heat capacity, which exhibits a peak at the Widom line that gradually fades with increasing pressure^47,61^.

This work seeks to contribute to the understanding of the diffusion behavior of binary ${\hbox {CO}}_{2}$ mixtures containing hydrocarbons in the extended critical region. To cover a broad range of solute size, methane, ethane, isobutane, benzene, toluene and naphthalene are considered in a systematic way. Building upon our recent work on thermodynamics, dynamics and structure of sc${\hbox {CO}}_{2}$ mixtures across the Widom line^47^, it focuses on the thermodynamic factor and diffusion coefficients. For this purpose, equilibrium molecular dynamics simulations were conducted to sample intra-diffusion, Maxwell–Stefan and Fick diffusion coefficients of those sc${\hbox {CO}}_{2}$ mixtures. The self-diffusion coefficients of the components within a mixture are commonly referred to as intra-diffusion coefficients, which helps to distinguish them from the self-diffusion coefficient of pure substances. Whenever possible, simulation data are compared with experiments and equation of state (EoS) models. An overview of the different diffusion coefficient types addressed in this work is given in Table 2. Further, new experimental data for ${\hbox {CO}}_{2}$ mixtures with benzene, toluene, n-dodecane and 1,2,3,4-tetrahydronaphthalene (THN) at high temperatures along the isobar *p* = 10 MPa are presented, cf. Table  3.

Molecular modeling and simulation is recognized as a powerful tool for predicting thermodynamic properties of ${\hbox {CO}}_{2}$ mixtures^62^. It provides valuable insights into the molecular-level interactions, structure and dynamics of fluids. For instance, such simulation studies have revealed significant clustering^63,64^ and the breakdown of the Stokes–Einstein relation in the vicinity of the Widom line^65,66^. However, conducting molecular simulations in the extended critical region poses challenges due to strong density fluctuations^55^, requiring a significant sampling effort to obtain statistically reliable results. Hence, only few molecular dynamics simulation studies have investigated diffusion coefficients of ${\hbox {CO}}_{2}$ mixtures with methane^56,67–70^, ethane^68^, benzene^71–74^, toluene^72,73^ or naphthalene^73–78^ at near-critical and/or supercritical states.

In this work, the Fick diffusion coefficient of sc${\hbox {CO}}_{2}$ mixtures with benzene, toluene, n-dodecane and THN at $p =$ 10 MPa was determined experimentally with the Taylor dispersion technique. This approach relies on the diffusive spreading of a small volume of a solution injected into a laminar stream of a carrier fluid. Axial dispersion spreads out the solute pulse longitudinally, while radial diffusion confines the pulse. Upon the flow of the mixture through a long capillary, a Gaussian concentration distribution, known as Taylor peak, is formed as a result of the combined effects of convective flow and molecular diffusion. Subsequently, the concentration distribution is detected at the end of the capillary. The Taylor dispersion technique has been employed since 1979^23^, but its accuracy still requires improvement for supercritical mixtures^79^. For instance, the solute is injected as a pure fluid into the ${\hbox {CO}}_{2}$ carrier, such that the diffusion process undergoes the entire composition range until it asymptotically reaches infinite dilution.

## Results

Mass transport in mixtures is commonly described by Fick’s “law”, which correlates the molar flux to the mole fraction gradient via the Fick diffusion coefficient. However, the actual thermodynamic driving force behind mass transport is the chemical potential gradient, which is balanced by friction forces between the mixture’s components as embodied by Maxwell–Stefan theory. This theory treats kinetics and thermodynamics separately, wherein the latter is given by the so-called thermodynamic factor $\Gamma$^80^. The kinetic contribution is considered by the Maxwell–Stefan diffusion coefficient Ɖ, which can be obtained from the phenomenological Onsager coefficients by molecular dynamics simulation. The Fick diffusion coefficient *D* can be determined experimentally. The ratio between these two diffusion coefficients is the thermodynamic factor, $\Gamma$ = *D*/Ɖ, which cannot be directly accessed with experiments.

In this study, six binary ${\hbox {CO}}_{2}$ mixtures were investigated close to the infinite dilution limit (typically with 0.5, 1.0 and 1.5 mol% of the solute) by molecular simulation. The extended critical region was in the focus, i.e., temperatures from 290 to 345 K along the isobar $p = 9$ MPa, and for ${\hbox {CO}}_{2}$ mixtures with the aromatics, additionally the isobars $p = 10$ and 12 MPa were considered. Taylor dispersion experiments of sc${\hbox {CO}}_{2}$ mixtures with benzene, toluene, n-dodecane and THN were also conducted at $p = 10$ MPa. Within the extended critical region, it was found that the thermodynamic factor, and consequently the Fick diffusion coefficient, exhibit a peculiar behavior.

**Table 1:** Experimental literature data for ${\hbox {CO}}_{2}$ mixtures with benzene, toluene or naphthalene at different temperatures and pressures.

Authors	Year	$N_{P}$	*T* / K	*p* / MPa	Experimental method
${\hbox {CO}}_{2}$ + Benzene					
Swaid and Schneider^23^	1979	22	313.15–328.15	11–15	TD
Sassiat et al.^12^	1987	11	303.15–333.15	11–26	TD
Uwezava and Nagashima^24^	1992	5	299.15–308.15	9–10.5	TD
Funazukuri et al.^18^	1992	2	313.2	16–25	TD
Levelt Sengers et al.^25^	1993	9	306.8–314.4	9.7–13.5	TD
Suarez et al.^26^	1993	15	313.15–333.15	15–35	TD
Funazukuri and Nishimoto^27^	1996	31	313.2	8.5–30	TD
Funazukuri et al.^28^	2001	176	308.15–328.15	6–30	TD
Filho et al.^29^	2002	7	313.15–333.15	12–16	TD
Nishiumi and Kubota^30^	2007	94	313.15	2.2–16.7	TD
Lin et al.^31^	2010	36	313.15–373.15	10–30	TD
Santos et al.^32^	2022	7	306.15–320.15	7.5–17.5	TD
This work	2023	7	302.8–341.5	10	TD
${\hbox {CO}}_{2}$ + Toluene					
Bruno^33^	1989	6	313	13.3–30.4	SFC
Levelt Sengers et al.^25^	1993	8	306.8–314.4	9.7–13.5	TD
Suarez et al.^26^	1993	15	313.15–333.15	15–35	TD
Lai and Tan^13^	1995	18	308–328	7.5–14.3	CCC
Cadogan^5^	2015	30	298–423	1–68	TD
Lin et al.^31^	2010	36	313.15–373.15	10–30	TD
Santos et al.^32^	2022	12	306.15–320.15	7.5–17.5	TD
This work	2023	8	302.9–341.5	10	TD
${\hbox {CO}}_{2}$ + Naphthalene					
Knaff and Schlünder^34^	1987	16	308.15–333.15	11.8–22.6	PSSDT
Sassiat et al.^12^	1987	11	303.15–333.15	11–26	TD
Lamb et al.^35^	1989	25	351.15	2–20	NMR-Bessel
Funazukuri et al.^18^	1992	2	313.2	16–25	TD
Akgerman et al.^36^	1996	17	308–328	9.6–24.9	TD
Klask^37^	1998	46	309–330.7	8.9–20	PSSDT
Higashi et al.^38^	1998	15	308.2	7.2–15.2	PSSDT
Higashi et al.^39^	1999	35	308.2–318.2	7.7–20	PSSDT
Higashi et al.^40^	2002	7	308.2	8.2–10.4	PSSDT
Kong et al.^41^	2011	42	308.15 and 333.15	7–40	TD

The number of data points is given by $N_{P}$. The accompanying numerical data are provided in the Supplementary Information.

The abbreviations for the experimental methods are TD, Taylor dispersion; SFC, Supercritical fluid chromatography; CCC, Coated capillary column, PSSDT, Pseudo-steady state solid dissolution technique and NMR-Bessel, NMR fixed field gradient spin-echo Bessel function analysis technique.

**Table 2:** Overview of the diffusion coefficient types adressed in this work.

Symbol	Name
$D_{\text {CO2}}$	Self-diffusion coefficient of pure ${\hbox {CO}}_{2}$
$D^{+}_{\text {CO2}}$	Scaled self-diffusion coefficient of pure ${\hbox {CO}}_{2}$
$D_{i}$	Intra-diffusion coefficient of component *i* in a mixture
Ɖ	Maxwell–Stefan diffusion coefficient
*D*	Fick diffusion coefficient
$D^{x_1\rightarrow 1}$	Infinite dilution Fick diffusion coefficient

### Thermodynamic factor

**Figure 1:**
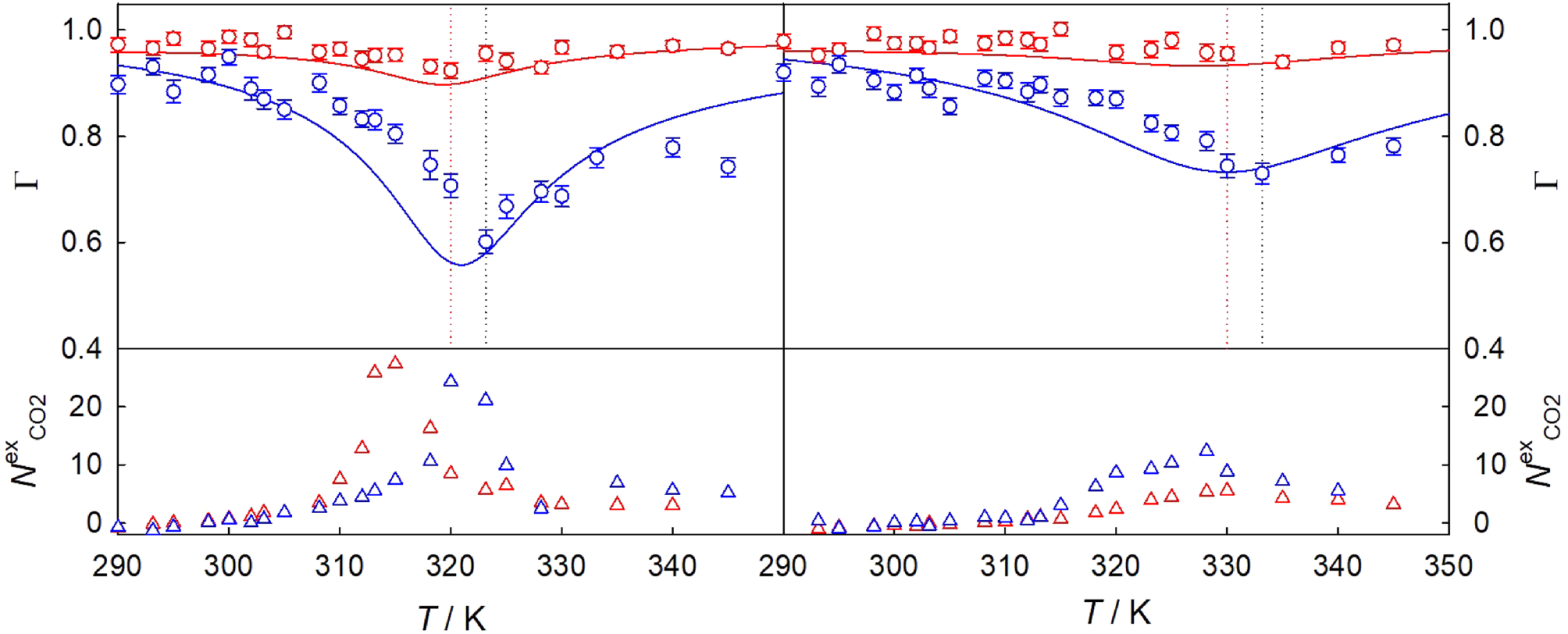
Temperature dependence of the thermodynamic factor (top) and the excess number of solvent molecules (bottom) of ${\hbox {CO}}_{2}$ mixtures with 0.5 mol% of benzene (red) or naphthalene (blue) along the isobars $p = 10$ MPa (left) and 12 MPa (right). Symbols represent molecular simulation data along the isobars $p =$ 10 and 12 MPa, respectively. Solid lines depict the thermodynamic factor calculated with TREND 5.0^83^ on the basis of the Peng–Robinson EoS for ${\hbox {CO}}_{2}$ + benzene ($k_{\text {12}}~= 0.0967$)^84^ or ${\hbox {CO}}_{2}$ + naphthalene ($k_{\text {12}}~= 0.016, l_{\text {12}}~= -0.173$)^40^. Dotted lines indicate the thermodynamic factor minimum inferred from molecular simulation data.

The thermodynamic factor $\Gamma = ({\partial \mu _1}/{\partial x_1})_{T,p} \cdot {x_1}/{(RT)}$, being the partial derivative of the chemical potential $\mu _1$ with respect to the mole fraction $x_1$, accounts for the non-ideality and is unity for ideal mixtures according to Raoult. Its dependence on temperature *T* and pressure *p* is exemplarily depicted for sc${\hbox {CO}}_{2}$ mixtures with 0.5 mol% of benzene or naphthalene in Fig. 1 (top). Plots on the temperature, pressure and mole fraction dependencies of $\Gamma$ for the remaining mixtures are given in Figs. S1 to S4 of the Supplementary Information (SI).

The thermodynamic factor, calculated by both Kirkwood-Buff integration and EoS, exhibits a consistent qualitative pattern characterized by an often pronounced minimum near the Widom line^57^. For example, the thermodynamic factor of sc${\hbox {CO}}_{2}$ containing only 0.5 mol% of naphthalene, reaches a value as low as $\Gamma = 0.45$ at $T = 320$ K along the isobar $p = 9$ MPa, cf. Fig. S1. Note that the magnitude of its minimum varies with the solute. In fact, as the size difference between the solvent and solute increases, the minimum becomes deeper, which aligns with the expected increase of the mixtures’ non-ideality.

In general, molecular simulation data sampled with Kirkwood-Buff integration tend to overestimate the thermodynamic factor when compared to the values obtained from EoS^81^. As the solute mole fraction goes to zero, $\Gamma \rightarrow 1$, cf. Figs. S1 to S4. Further, the thermodynamic factor minimum at a given mixture composition fades with rising pressure and shifts to higher temperatures, cf. Fig. 1.

The presence of that minimum is attributed to the proximity of such states to the critical point, where $\Gamma = 0$^82^. In fact, the observed strong sensitivity of the thermodynamic factor to even small changes of solute concentration^30^ and the occurrence of clustering^63^ can be related to this proximity. The extent of clustering can be quantified by the excess number of solvent molecules around a given solute molecule, relative to a uniform bulk density distribution^47,64^. Consequently, the presence of clustering may result in a negative partial molar volume of the solute component near the infinite dilution limit^47^.

Fig. 1 (bottom) illustrates the dependence of the excess coordination number on temperature and pressure for sc${\hbox {CO}}_{2}$ mixtures with benzene and naphthalene along the isobars $p =$ 10 and 12 MPa. The temperature where thermodynamic non-idealities are most significant, indicated by the minimum of $\Gamma$, approximately coincides with the one where the largest number of solvent molecules gathers around a given solute molecule. This suggests that clustering on the molecular scale is responsible for the observed non-idealities.

### Fick diffusion coefficient

The Maxwell–Stefan and intra-diffusion coefficients were sampled with molecular dynamics simulation at three solute mole fractions (typically 0.5, 1.0 and 1.5 mol%) in the regarded temperature and pressure range. Using Kirkwood-Buff integration simulation data for the thermodynamic factor, the Fick diffusion coefficient was obtained from *D* = Ɖ$\cdot \Gamma$. In the infinite dilution limit, Fick and Maxwell–Stefan diffusion coefficients coincide, thus both can be estimated by the extrapolation of the intra-diffusion coefficient of the solute (2) to the limit where it is infinitely diluted in ${\hbox {CO}}_{2}$ solvent (1)^56,85^. This extrapolation of the intra-diffusion coefficient is associated with lower statistical uncertainties than the directly sampled Maxwell–Stefan diffusion coefficient, since the latter is a collective property.

**Figure 2:**
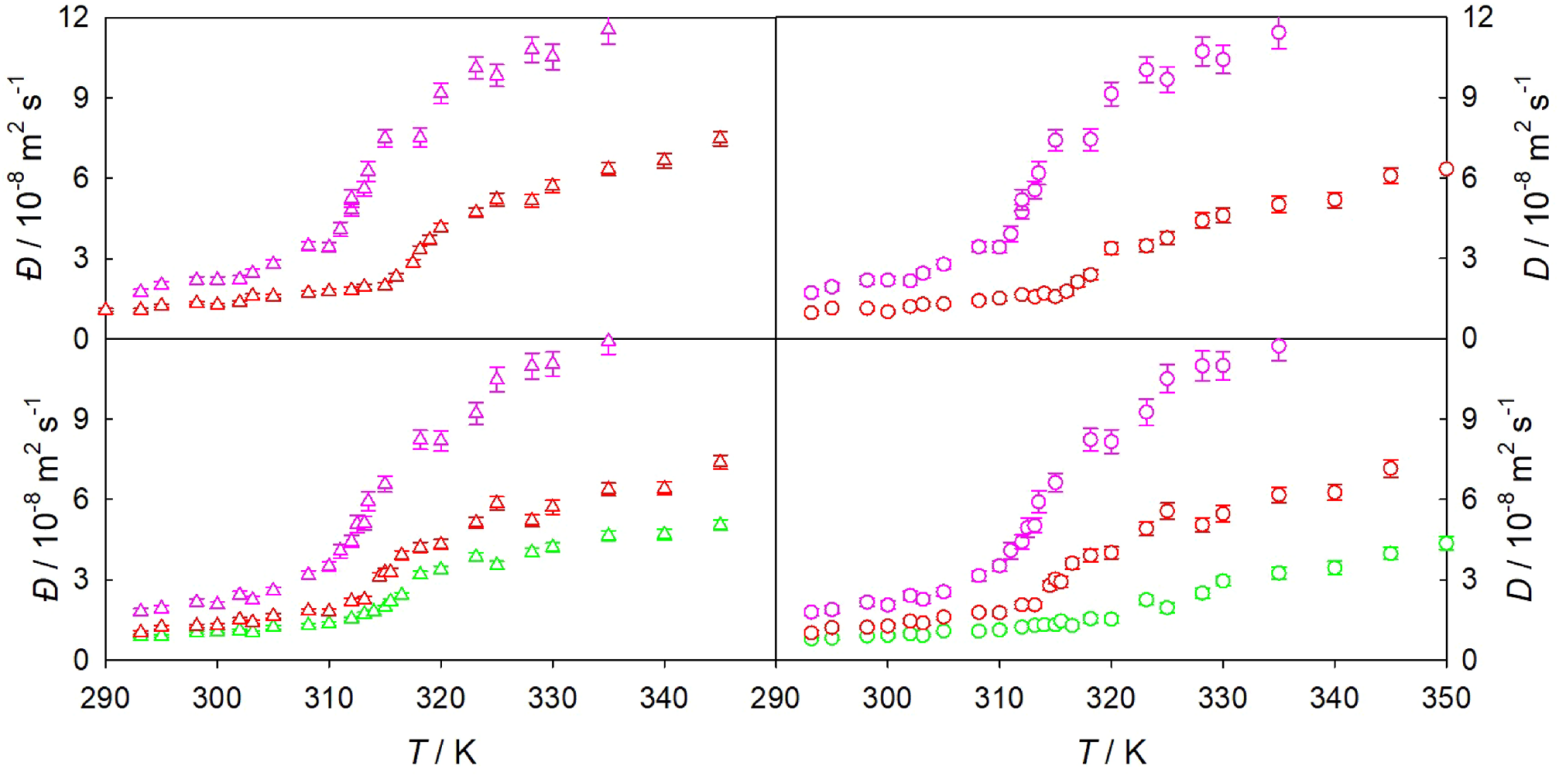
Temperature dependence of the Maxwell–Stefan (left) and Fick diffusion (right) coefficients of ${\hbox {CO}}_{2}$ mixtures with 0.5 mol% (bottom) and 1.5 mol% (top) of ethane (pink), benzene (red) or naphthalene (green) along the isobar $p = 9$ MPa. Symbols represent molecular simulation data.

The influence of solute mole fraction on the Maxwell–Stefan diffusion coefficient was found to be weak, as illustrated in Fig. 2 or Fig. S5. In contrast, the Fick diffusion coefficient depends significantly on composition in the critical region, which can be attributed to substantial changes of the thermodynamic factor. Usually, both the Maxwell–Stefan and Fick diffusion coefficients increase with rising temperature. However, the Fick diffusion coefficient may decrease near the Widom line as the temperature rises due to a significant reduction of the thermodynamic factor.

#### Density dependence

**Figure 3:**
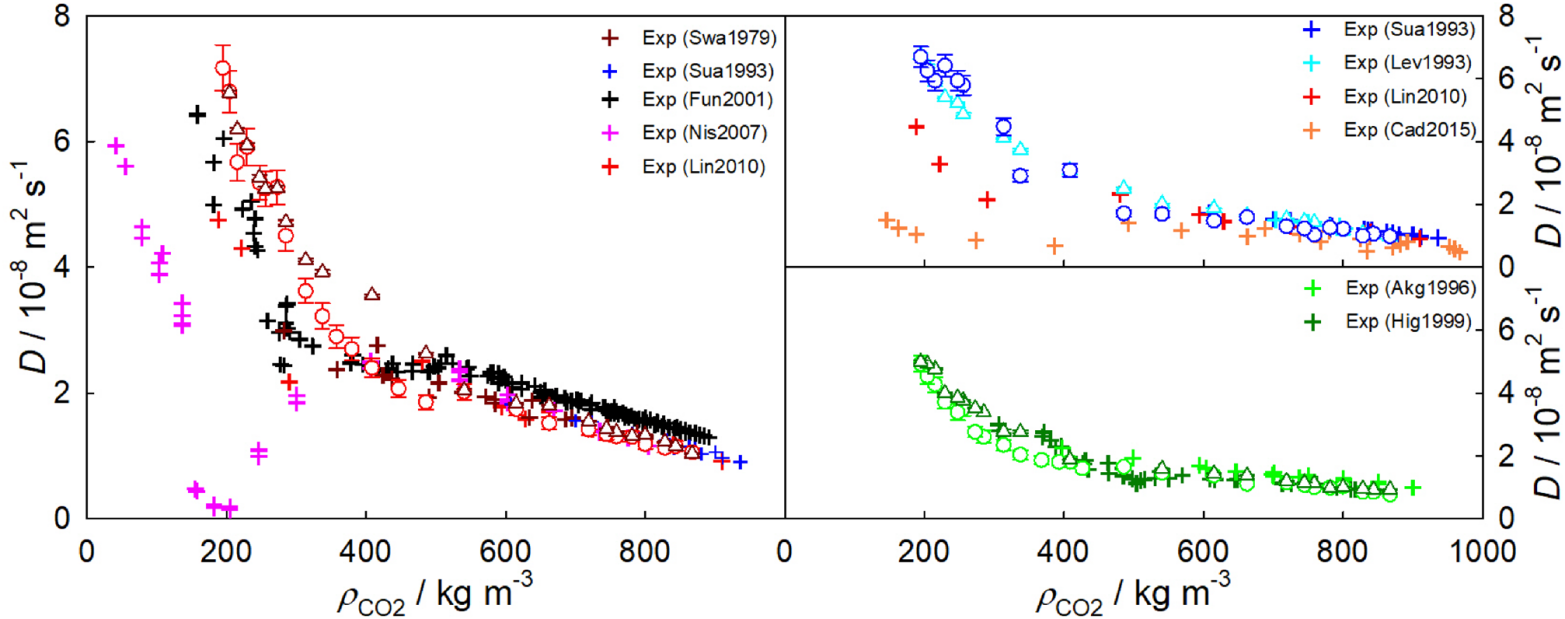
Density dependence of the Fick diffusion coefficient of ${\hbox {CO}}_{2}$ mixtures with benzene (left), toluene (top right) or naphthalene (bottom right). Open circles represent molecular simulation data for a solute mole fraction of 1.0 mol% for benzene or toluene and of 0.3 mol% for naphthalene. Open triangles represent the infinite dilution Fick diffusion coefficient along the isobar *p* = 9 MPa. Experimental data for benzene^23,26,28,30,31^ at *p* = 2.2 to 35 MPa, toluene^5,25,26,31^ at *p* = 1 to 68 MPa and naphthalene^36,39^ at *p* = 2 to 40 MPa are depicted by crosses. Further experimental data are listed in Table 1, but were left out in the plot to avoid visual clutter.

The density dependence of the Fick diffusion coefficient of ${\hbox {CO}}_{2}$ mixtures with the aromatics benzene, toluene or naphthalene is shown in Fig. 3. To facilitate a more straightforward comparison of data from different sources, present simulation results were compared to experimental data of mixtures having the same density, but may have been measured at different temperatures or pressures, cf. Table 1.

In general, a good qualitative agreement between experimental literature and present simulation data is found. However, it is evident that the experimental data scatter significantly at near-critical densities. As expected, the Fick diffusion coefficient gradually rises with decreasing density, but the presence of a peculiar behavior of that coefficient close to the Widom line can be inferred. For instance, either the Fick diffusion coefficient exhibits a minimum (red crosses in Fig. 3) or the rate of its decrease with rising density is slowed down in certain state regions (black crosses in Fig. 3). This is confirmed by present experiments and is most pronounced for large and heavy solutes, such as n-dodecane or THN, which show a stronger drop of the Fick diffusion coefficient in the vicinity of the Widom line than benzene or toluene, cf. Table 3. These experimental findings can be attributed to a stronger departure from ideality of the mixture, leading to a more pronounced thermodynamic factor minimum^30^. In contrast to molecular simulation runs, the exact solute mole fraction is unknown in most diffusion coefficient experiments. An exception are the Fick diffusion coefficient data for ${\hbox {CO}}_{2}$ + benzene from Nishiumi und Kubota^30^, which were measured at a solute mole fraction of approximately 1.7 mol%^86^.

The quantitative agreement between data from experiment and simulation is good at high densities and low temperatures along the isobar $p =$ 9 MPa. The peculiar behavior of the experimentally determined Fick diffusion coefficient is qualitatively replicated by simulation data at finite mole fractions. The thermodynamic factor has a minimum close to the Widom line, and consequently decreases the Fick diffusion coefficient in the near-critical region. This strong concentration dependence of that coefficient^87^ and its peculiar behavior fade in the infinite dilution limit, where the thermodynamic factor converges to unity. Hence, the thermodynamic factor is a key property to rationalize diffusion in SCF mixtures. In fact, the thermodynamic factor has been demonstrated to cause the strong reduction of the experimental Fick diffusion coefficient of ${\hbox {CO}}_{2}$ + naphthalene even for a solute mole fraction as low as 0.1 mol%^40,86^.

The strong variations of density and thus of the diffusion coefficients are most pronounced at the lowest isobar $p =$ 9 MPa, where the mixtures are closer to the critical point of ${\hbox {CO}}_{2}$. As the pressure increases, the peculiar behavior of both the thermodynamic and transport properties fades^47^. Further, the infinite dilution Fick diffusion coefficient decreases at constant temperature with rising pressure.

**Figure 4:**
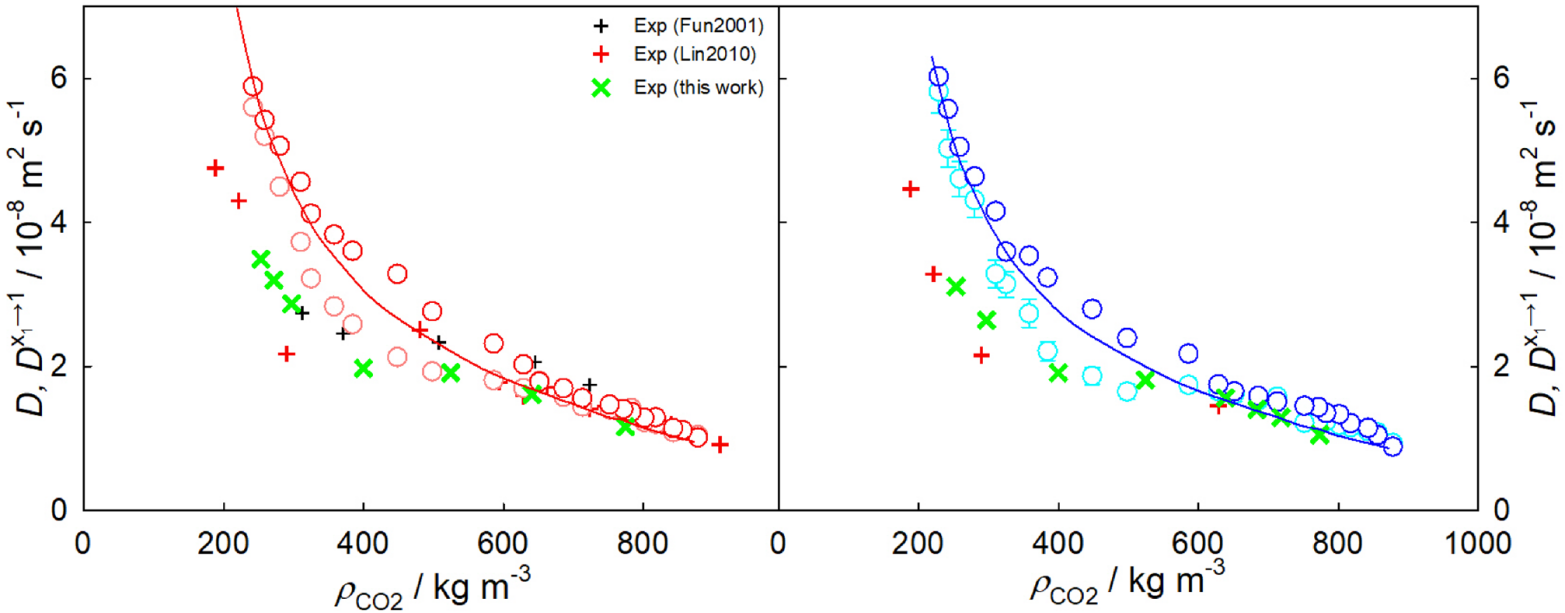
Density dependence of the Fick diffusion coefficient of ${\hbox {CO}}_{2}$ mixtures with benzene (left) or toluene (right) along the isobar $p =$ 10 MPa. Circles represent molecular simulation data at a solute mole fraction of 1.5 mol% for benzene (pink) or toluene (cyan). The infinite dilution Fick diffusion coefficient $D^{x_1\rightarrow 1}$ of ${\hbox {CO}}_{2}$ + benzene and ${\hbox {CO}}_{2}$ + toluene is depicted by red and blue circles, respectively. Experimental data from this work are shown by green crosses, whereas literature data are represented by black^28^ and red^31^ plus signs. Solid lines show the predictive approach proposed in this work (Eqs. (2) and (4)). Additional experimental data are listed in Table 1, but were left out of the plot to avoid visual clutter.

A comparison of present experimental data with simulation data for sc${\hbox {CO}}_{2}$ mixtures with benzene or toluene at $p =$ 10 MPa is given in Fig. 4. There, molecular simulation overestimates the experimental Fick diffusion coefficient at low densities for both mixtures, but the qualitative agreement is good. The peculiar behavior of the Fick diffusion coefficient in the near-critical region is both qualitatively and quantitatively replicated at finite mole fractions.

#### Solute mass dependence

Previous experimental studies have demonstrated that the infinite dilution Fick diffusion coefficient of 44 sc${\hbox {CO}}_{2}$ mixtures is logarithmically correlated with the mass of the solute^14,43,88^. This suggests that the molecular weight of the solute may have a greater impact on mutual diffusion in diluted sc${\hbox {CO}}_{2}$ mixtures than the molecular interactions^14^.

**Figure 5:**
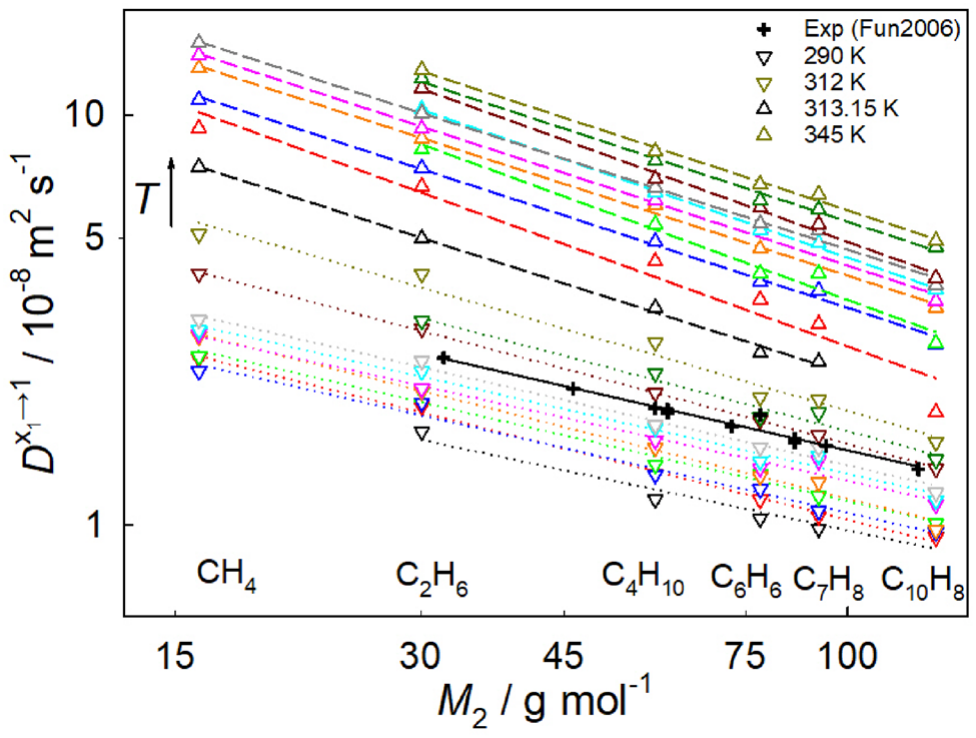
Molecular solute mass dependence of the infinite dilution Fick diffusion coefficient of ${\hbox {CO}}_{2}$ mixtures with methane, ethane, isobutane, benzene, toluene or naphthalene. Symbols represent molecular simulation data for different temperatures along the isobar *p* = 9 MPa: triangles down for $T = 290$ to 312 K and triangles up for $T = 313.15$ to 345 K. Crosses represent experimental literature data^43^ for other solutes (ranging from methanol to benzoic acid) in sc${\hbox {CO}}_{2}$ at $T = 313.2$ K and $p = 11$ MPa. Dotted and dashed lines represent the liquid- and gas-like regions of pure ${\hbox {CO}}_{2}$, respectively, which are linear regressions of the form $\log (D^{x_1\rightarrow 1}) \sim \log (M_2).$

In the present study, the dependence of the infinite dilution Fick diffusion coefficient on molecular solute mass was investigated for six hydrocarbons infinitely diluted in sc${\hbox {CO}}_{2}$ at 22 thermodynamic state points along the isobar *p* = 9 MPa, cf. Fig. 5. The simulation results validate the trends observed in previous experimental studies. For all mixtures, the infinite dilution Fick diffusion coefficient increases with rising temperature. The slope of that function is consistent within the high-density, liquid-like ($T<$ 308.15 K, dotted lines) and the low-density, gas-like ($T>$ 315 K, dashed lines) SCF regions. At the limits of the regarded temperature range, the curves exhibit a noticeable parallelism, which weakens as the Widom line is approached. In fact, there is a notable 27% change in slope between both density regions when crossing the Widom line of pure ${\hbox {CO}}_{2}$, i.e. from $\sim M_2^{-0.528}$ at $T = 308.15$ K to $\sim M_2^{-0.719}$ at $T = 315$ K. Thus, the solute mass dependence of the infinite dilution Fick diffusion coefficient varies significantly across distinct density regions of supercritical fluids.

### Breakdown of Stokes–Einstein relation

The Stokes–Einstein (SE) relation establishes a connection between the diffusive motion of a solute with its size and the surrounding fluid’s viscosity. In this work, the SE equation $D_i = k_B T/(C \eta r_{H,i}$) was applied to the intra-diffusion coefficients of the mixtures’ components $D_i$. Therein, $r_{H,i}$ is the hydrodynamic radius of component *i*, $\eta$ is the shear viscosity of the fluid, $k_B$ is the Boltzmann constant and *C* accounts for the boundary conditions. For slip conditions, $C = 4 \pi$, whereas for stick conditions, $C = 6 \pi$. This relation assumes that the hydrodynamic radius $r_{H,i}$ is constant and independent of temperature^89^, which implies that $D_i {\eta }/T$ is temperature-independent^65^.

Several experimental studies have indicated that diffusion in sc${\hbox {CO}}_{2}$ mixtures closely follows the hydrodynamic behavior expected from the SE relation, i.e. $D_i \propto T/\eta$^31,44,45^, but its validity was questioned ever since^66,90–92^. The region near the Widom line, where a transition from a high-density to a low-density fluid and a sharp growth of molecule clusters occur, has been found to be correlated to the strong decoupling of diffusion and viscosity, i.e. the breakdown of the SE relation, e.g., for water^65^.

There are different methods to verify the validity of the SE relation^93–95^. One popular approach involves the calculation of the hydrodynamic radius $r_{H,i}$, but this requires the knowledge of the boundary conditions and shear viscosity. To avoid issues related to the accuracy of these values, the ratio

1 $$\begin{aligned} \frac{D_2}{D_1} = \frac{k_B T/({r_\text {H,2}} \eta C)}{k_B T/({r_{\text {H,1}}} \eta C)} = \frac{r_{\text {H,1}}}{r_{\text {H,2}}}, \end{aligned}$$

can be considered, where $D_2$ and $D_1$ are the intra-diffusion coefficients of the solute and ${\hbox {CO}}_{2}$, respectively.

**Figure 6:**
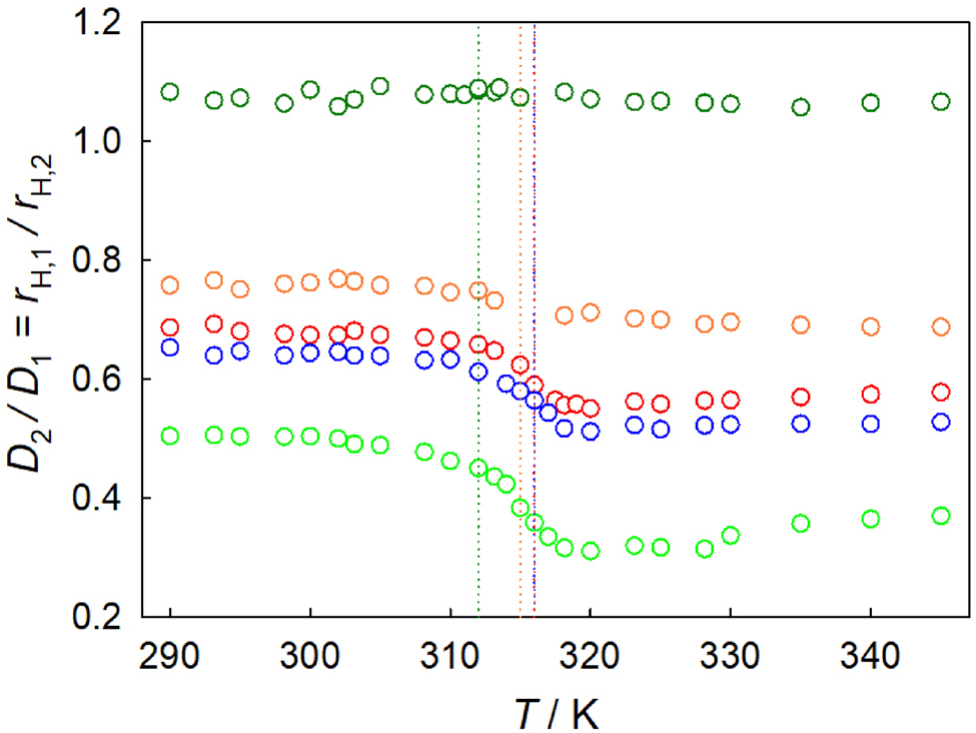
Ratio of solute $D_2$ to solvent $D_1$ intra-diffusion coefficients of ${\hbox {CO}}_{2}$ mixtures with 1.5 mol% of ethane (dark green), isobutane (orange), benzene (red), toluene (blue) or with 0.6 mol% of naphthalene (green). Symbols represent simulation data along the isobar $p = 9$ MPa. Dotted lines indicate the Widom line temperature of the respective ${\hbox {CO}}_{2}$ mixture inferred from molecular simulation data.

The ratio of intra-diffusion coefficients of sc${\hbox {CO}}_{2}$ mixtures along the isobar *p* = 9 MPa is shown in Fig. 6. As expected from the SE relation, the ratio of hydrodynamic radii remains relatively constant in the high-density, liquid-like region up to the Widom line. There, a significant change of this ratio is observed for several of the studied mixtures, which suggests a breakdown of the SE relation. At higher temperatures, in the low-density, gas-like region, the ratio again follows the expected SE behavior to some extent. It should be noted that the magnitude of the SE breakdown increases with the depth of the thermodynamic factor minimum, thus being related to the non-ideality. A rising pressure weakens this breakdown due to the reduction of fluctuations and clustering related to the critical region^90^. The breakdown of the SE relation implies a change of the predominant diffusion mechanisms around the Widom line, which is associated with an increase of the translational jump-diffusion of molecules with large-amplitude displacements. This phenomenon has been observed in aqueous alcoholic mixtures^91,96^.

However, the breakdown of the SE relation is much weaker for ${\hbox {CO}}_{2}$ + isobutane and may hardly be discerned for ${\hbox {CO}}_{2}$ + ethane. This can be explained with significantly less pronounced clustering, which is associated with a much weaker non-ideality of those mixtures, due to the relative similarity of these molecular species in comparison to ${\hbox {CO}}_{2}$ + aromatics^47^.

### Semi-empirical correlations

Eleven correlations for the estimation of the infinite dilution Fick diffusion coefficient of sc${\hbox {CO}}_{2}$ mixtures as listed in Table S1 were assessed. These correlations include the SE-based equations by Wilke and Chang^8^, Scheibel^9^, Tyn and Calus^10^, Hayduk and Minhas^11^, Sassiat^12^, Lai and Tan^13^, the hydrodynamic equation (modified SE)^14,31^ and the modified Rice-Gray correlation^15^, as well as the free-volume based equations by Catchpole and King^16^, He and Yu^17^ and Funazukuri and Wakao^97^.

**Figure 7:**
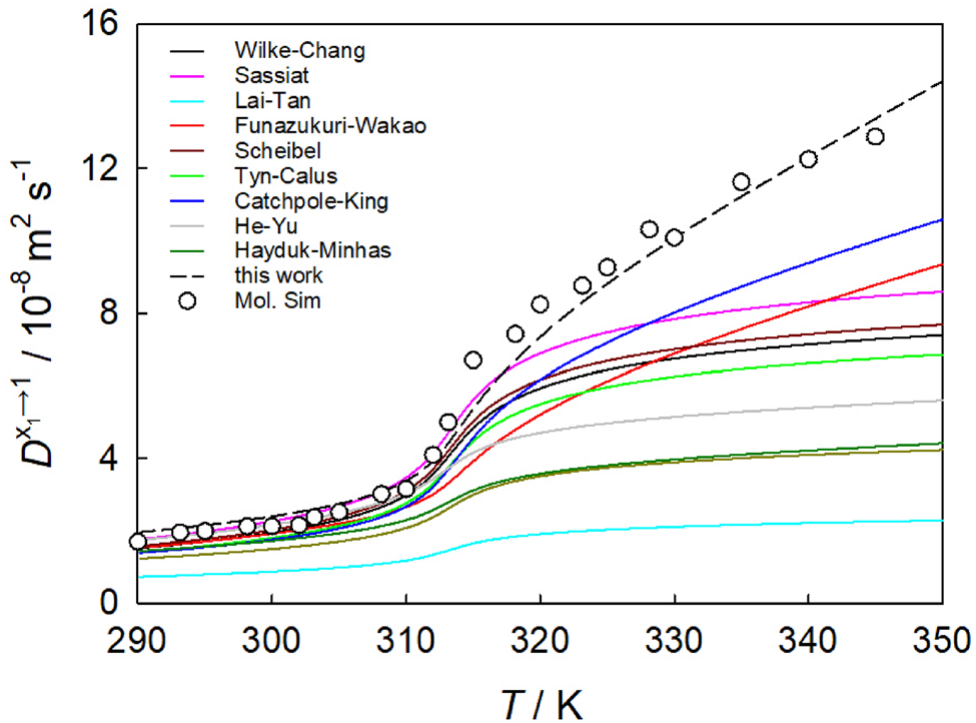
Temperature dependence of the infinite dilution Fick diffusion coefficient of ${\hbox {CO}}_{2}$ + ethane along the isobar *p* = 9 MPa. Circles represent molecular simulation data obtained from the extrapolation of the intra-diffusion coefficient of ethane to the infinite dilution limit. Solid lines depict semi-empirical correlations^8–14,16–18^ and the dashed line shows the present predictive approach introduced later in the manuscript (Eqs. (2) and (4)).

This set of correlations was compared to simulation data for all studied thermodynamic conditions, cf. Fig. 7 and Figs. S6 to S10 of the SI. The average absolute relative deviation (AARD) between these correlations and present simulation data is listed in Table S1 of the SI. Overall, simulation data exhibit a good qualitative agreement with the correlations. The Wilke–Chang and Catchpole–King equations demonstrate the best overall quantitative agreement for the studied ${\hbox {CO}}_{2}$ + hydrocarbon mixtures, with an AARD of 13.0% and 13.3%, respectively. Although the modified Rice-Gray correlation^15^ has a more complex form, it deviates on average by 8.3% from simulation data, compared to the 7.7% and 8.4% of the Wilke–Chang and Tyn–Calus correlations, respectively, for the mixtures of ${\hbox {CO}}_{2}$ with aromatics at all considered pressures. On the other hand, the He-Yu equation^17^ is very effective at temperatures below the Widom line, i.e., in the liquid-like SCF, with a deviation of only 6.1% from the simulation data in that region.

At temperatures above the Widom line, i.e., in the gas-like SCF state, the agreement between simulation and the correlations deteriorates significantly. None of the correlations accurately predicts the sampled Fick diffusion coefficient data across the entire range of supercritical conditions. This observation holds also when comparing the correlations with the experimental data from this work for ${\hbox {CO}}_{2}$ mixtures with benzene or toluene at a pressure of 10 MPa. Most correlations show an AARD of at least 20% from present experimental data, cf. Table S1. Exceptions are the correlations by Funazukuri-Wakao^18^ and He-Yu^17^, which predict an infinite dilution Fick diffusion coefficient that shows an AARD of approximately 10.8%.

### Infinite dilution Fick diffusion coefficient

**Figure 8:**
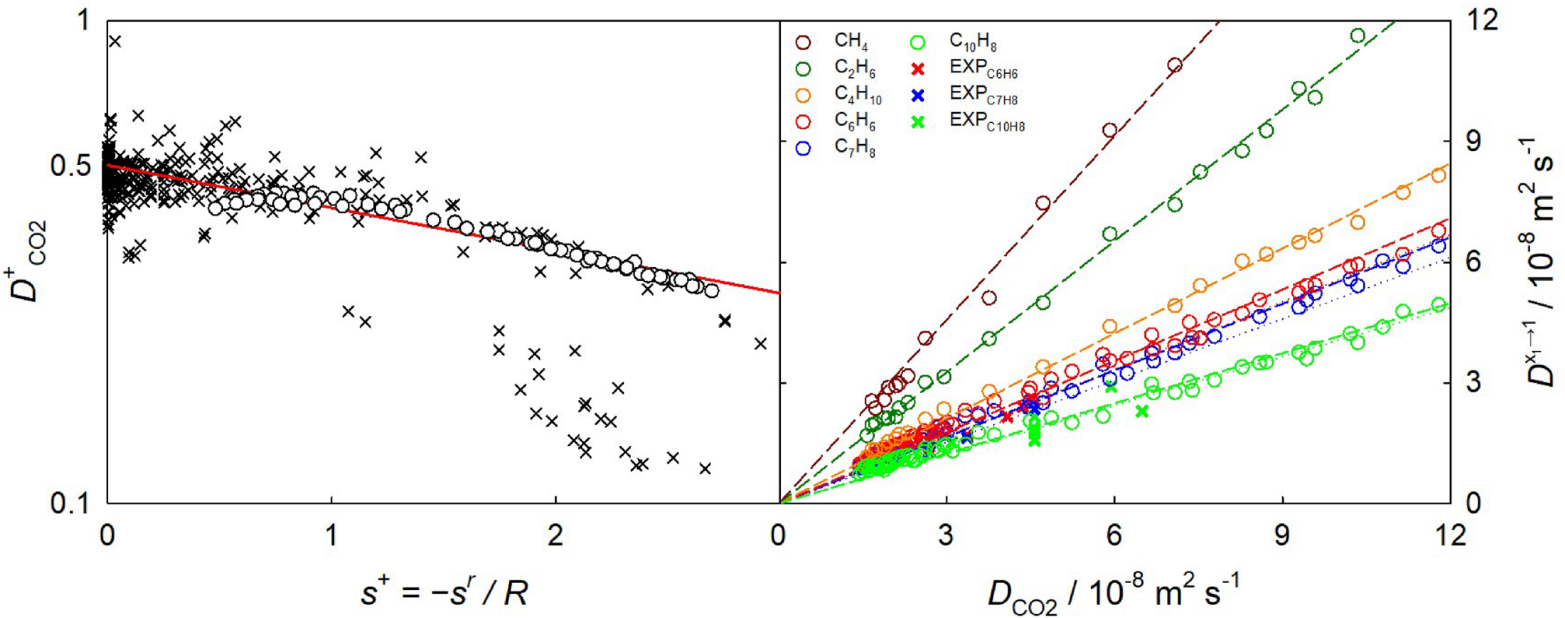
Self-diffusion coefficient of pure ${\hbox {CO}}_{2}$ scaled according to Bell et al.^100^ over the reduced residual entropy (left). Experimental data for the ${\hbox {CO}}_{2}$ self-diffusion coefficient^103^ and simulation data are represented by crosses and circles, respectively. The solid line (red) depicts the present entropy scaling correlation $D^{+}_{\text {CO2}} = 0.5016 \cdot \exp {(-0.2031 \cdot s^{+})}$. On the right, the infinite dilution Fick diffusion coefficient of ${\hbox {CO}}_{2}$ mixtures is shown as a function of the self-diffusion coefficient of pure ${\hbox {CO}}_{2}$. Simulation data for ${\hbox {CO}}_{2}$ mixtures with methane (dark red), ethane (dark green), isobutane (orange), benzene (red), toluene (blue) or naphthalene (green) are represented by open circles. Experimental data for the infinite dilution Fick diffusion coefficient of ${\hbox {CO}}_{2}$ with benzene, toluene and naphthalene (right) are depicted by crosses. Linear regressions of the simulation data are shown by dashed lines.

A two-step approach to predict the infinite dilution Fick diffusion coefficient of arbitrary ${\hbox {CO}}_{2}$ mixtures on the basis of temperature, pressure (or density) and solute mass is proposed, cf. Fig. 8. In a first step, the self-diffusion coefficient of pure ${\hbox {CO}}_{2}$ is estimated with an entropy scaling approach. Subsequently, this coefficient $D_{\text {CO2}}$ is related to the infinite dilution Fick diffusion coefficient by a linear function of molecular solute mass.

First, the Span-Wagner EoS^98^, that is freely available at the NIST Webbook^99^, can be used to estimate the reduced residual entropy $s^{+} = -s^r / R$ of ${\hbox {CO}}_{2}$ at the specified temperature and pressure (or density). The following correlation relates the self-diffusion coefficient of pure ${\hbox {CO}}_{2}$ (in m$^2$/s) with the reduced residual entropy

2 $$\begin{aligned} D_{\text {CO2}} = \frac{1}{(\rho N_A)^{1/3} ({M_1/(RT)})^{1/2}} \cdot \frac{0.5016 \cdot \exp {(-0.2031 \cdot s^{+})}}{(s^+)^{2/3}}, \end{aligned}$$

where $\rho$ the molar density in mol/m$^3$, $N_A$ the Avogadro constant, $M_1$ the molecular solvent mass in kg/mol, *R* the universal gas constant in J/(mol K) and *T* the temperature in K. This equation can also be expressed in the following form

3 $$\begin{aligned} D^{+}_{\text {CO2}} = D_{\text {CO2}} \cdot (\rho N_A)^{1/3} ({M_1/(RT)})^{1/2} \cdot (s^+)^{2/3} = 0.5016 \cdot \exp {(-0.2031 \cdot s^{+})}, \end{aligned}$$

where $D^{+}_{\text {CO2}}$ is the self-diffusion coefficient of ${\hbox {CO}}_{2}$ scaled according to Bell et al.^100^. The predicted self-diffusion coefficient shows an AARD of $\sim$ 2.4% to the underlying simulation data depicted in Fig. 8 (left).

Second, present simulations and experimental data indicate that the infinite dilution Fick diffusion coefficient of ${\hbox {CO}}_{2}$ mixtures with a given solute $D^{x_1\rightarrow 1}$ is a linear function of the self-diffusion coefficient of pure ${\hbox {CO}}_{2}$ under the studied thermodynamic conditions, cf. Fig.  8 (right). Since the linear equations for each of the solutes pass through the origin, they only differ by the slope, which, in turn, depends on the molecular mass of the solute $M_2$ by

4 $$\begin{aligned} D^{x_1\rightarrow 1} = 0.1276 \cdot {M_2}^{-0.6124} \cdot D_{\text {CO2}}. \end{aligned}$$

Hence, using Eqs. (2) and (4) yields the infinite dilution Fick diffusion coefficient of an arbitrary ${\hbox {CO}}_{2}$ mixture at the specified supercritical state point.

The entropy scaling approach by Bell et al.^100^ was applied in this work to estimate the self-diffusion coefficient of ${\hbox {CO}}_{2}$. This particular approach was chosen over Rosenfeld’s entropy scaling for liquids^101^ and dilute gases^102^, as it was demonstrated to successfully bridge the gap between these two aggregation states^100^.

Figure 8 (left) shows the self-diffusion coefficient $D^{+}_{\text {CO2}}$ scaled according to Bell et al. in combination with the Span-Wagner EoS in comparison with experimental^103^ and simulation data for pure ${\hbox {CO}}_{2}$. The simulation data exhibit an excellent agreement with the experimental data within the reduced residual entropy range $0.5<s^+<2.8$, which also validates the employed ${\hbox {CO}}_{2}$ force field model^104^. These data can also be represented in the scaled form proposed by Rosenfeld^101^ or by using the entropy-scaling coordinate^105,106^ as shown in Fig. S11. Further, other entropy-scaling based correlations to estimate the self-diffusion coefficient of ${\hbox {CO}}_{2}$ on the basis of input data from perturbed-chain polar statistical associating fluid theory (PCP-SAFT)^107,108^, translated-consistent Peng–Robinson (tc-PR) or industrialized PC-SAFT (I-PC-SAFT) EoS^106^ can also be used in the first step of the present approach. For instance, the correlation by Dehlouz et al.^106^ was recently used to estimate the self-diffusion coefficient of ${\hbox {CO}}_{2}$ and 71 other pure substances.

**Figure 9:**
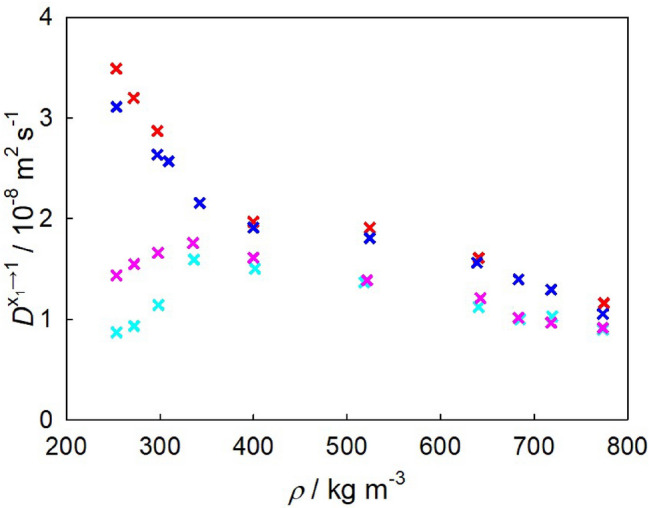
Present experimental data for the infinite dilution Fick diffusion coefficient as a function of density. Crosses represent data for ${\hbox {CO}}_{2}$ mixtures with benzene (red), toluene (blue), n-dodecane (pink) and THN (cyan) along the isobar *p* = 10 MPa.

The proposed two-step prediction approach yields an AARD of 7.4 % to simulation data for all ${\hbox {CO}}_{2}$ mixtures considered in this work, cf. Table S1 and Figs. S6 to S10. The deviation to present experimental data represented in Fig. 9 is 26.8%, which reduces to 11.8% if only the density region $\rho _{\text {CO2}} > 400$ kg/m$^3$ is considered.

## Discussion and conclusion

Molecular dynamics simulations were complemented by Taylor dispersion experiments, equations of state and semi-empirical correlations to study the Fick diffusion coefficient of binary ${\hbox {CO}}_{2}$ mixtures in the extended critical region, i.e. in the vicinity of the Widom line. An overview of the available experimental data on the Fick diffusion coefficient of hydrocarbons diluted in sc${\hbox {CO}}_{2}$ was presented together with new experimental data for sc${\hbox {CO}}_{2}$ mixtures with benzene, toluene, n-dodecane and THN along the isobar $p =$ 10 MPa.

It was demonstrated that the thermodynamic factor exhibits a minimum in the proximity of the Widom line. Its magnitude depends on the non-ideality of the mixture, which is related to the size difference between ${\hbox {CO}}_{2}$ and the solute. This non-ideality is a consequence of density fluctuations and the formation of clusters comprising ${\hbox {CO}}_{2}$ molecules around a given solute molecule. The extent of this clustering was quantified by means of the excess coordination number.

The peculiarity of the Fick diffusion coefficient in the near-critical region can be attributed to the presence of a thermodynamic factor minimum. This phenomenon was shown to be particularly pronounced for large solute molecules, like naphthalene or n-dodecane. Consequently, the thermodynamic factor of these mixtures exhibits a more pronounced minimum due to the size disparity leading to stronger clustering.

In the infinite dilution limit, where the thermodynamic factor approaches unity, the Fick diffusion coefficient has a conventional behavior, i.e. it monotonously increases with falling density. As the pressure rises and the system moves further away from the critical region, the thermodynamic factor minimum fades, resulting in a gradual reduction of the peculiar behavior of the Fick diffusion coefficient at finite mole fractions.

A breakdown of the Stokes–Einstein relation was confirmed for the investigated ${\hbox {CO}}_{2}$ mixtures in the proximity of the Widom line. It is very notable for ${\hbox {CO}}_{2}$ mixtures with benzene, toluene or naphthalene, while it is less pronounced e.g. for ${\hbox {CO}}_{2}$ + ethane. The extent of this breakdown can be related to the non-ideality of the mixtures and the strong density fluctuations near the Widom line.

Eleven semi-empirical correlations to estimate the infinite dilution Fick diffusion coefficient of supercritical ${\hbox {CO}}_{2}$ mixtures were compared to experiments and simulations. Most of these correlations fail to predict the Fick diffusion coefficient under gas-like SCF conditions, characterized by high temperature and low density. The Wilke-Chang and Catchpole-King equations were found to have the best quantitative agreement with simulation data for the studied ${\hbox {CO}}_{2}$ mixtures, with overall AARD of 13.0% and 13.3%, respectively. On the other hand, the best agreement with the present experimental results for ${\hbox {CO}}_{2}$ mixtures with benzene or toluene was observed for the Funazukuri-Wakao and He-Yu correlations, having an average deviation of about 10.8%.

A predictive approach for the infinite dilution Fick diffusion coefficient of ${\hbox {CO}}_{2}$ + hydrocarbon mixtures on the basis of temperature, pressure (or density) and solute mass was proposed. In a first step, entropy scaling was employed to determine the self-diffusion coefficient of pure ${\hbox {CO}}_{2}$. Subsequently, this coefficient was used to determine the infinite dilution Fick diffusion coefficient of the mixture. These two diffusion coefficients exhibit a linear relationship, where the slope is solely dependent on solute mass. Compared to simulation data, this approach demonstrates an overall AARD of 7.4% for the ${\hbox {CO}}_{2}$ mixtures considered in this work, and hence, a significant improvement over semi-empirical correlations. However, when compared to present experimental data for ${\hbox {CO}}_{2}$ mixtures with benzene, toluene, n-dodecane and THN, it shows an AARD of 26.8%. This can be attributed to the use of simulation data for parameterization, which accurately predict the experimental Fick diffusion coefficient of ${\hbox {CO}}_{2}$ mixtures with aromatics under high-density, liquid-like conditions, but tend to overestimate it under low-density, gas-like conditions.

It should be noted that molecular simulation allows for the determination of the Fick diffusion coefficient for a specified mixture composition. In most experiments, the mixture composition strongly varies until it reaches the infinite dilution limit. In fact, the injected solute in the ${\hbox {CO}}_{2}$ carrier fluid is often injected as a pure fluid at the beginning of the diffusive process. This process thus undergoes the entire composition space. Upon the flow of the mixture through a long capillary, the pulse dilutes itself and the diffusive process approaches infinite dilution asymptotically. Therefore, Taylor dispersion experiments do not occur in the exact infinite dilution limit. When a single Fick diffusion coefficient is assigned to characterize this entire process, which may be slowed down because of the depression of the thermodynamic factor, a too low Fick diffusion coefficient would be identified. This may partially explain the quantitative discrepancies between data from experiments and simulation under low-density, gas-like conditions. Hence, the peculiarity, i.e. the depression of the Fick diffusion coefficient in the near critical region, may be rationalized with the strong composition dependence of this property.

It is expected that the presented two-step approach to predict the infinite dilution Fick diffusion coefficient can be extended to arbitrary ${\hbox {CO}}_{2}$ + hydrocarbon mixtures in the extended critical region. Further studies are needed to assess its applicability to ${\hbox {CO}}_{2}$ mixtures with associating molecular species.

**Table 3:** Present experimental data for ${\hbox {CO}}_{2}$ mixtures with benzene, toluene, n-dodecane and THN along the isobar $p = 10$ MPa. The numbers in parentheses indicate the uncertainties in last digits.

${\hbox {CO}}_{2}$ + Benzene		${\hbox {CO}}_{2}$ + Toluene		${\hbox {CO}}_{2}$ + n-Dodecane		${\hbox {CO}}_{2}$ + THN	
*T* / K	*D* / 10$^{-8}$ m$^2$ s$^{-1}$	*T* / K	*D* / 10$^{-8}$ m$^2$ s$^{-1}$	*T* / K	*D* / 10$^{-8}$ m$^2$ s$^{-1}$	*T* / K	*D* / 10$^{-8}$ m$^2$ s$^{-1}$
302.9	1.159 (20)	303.0	1.053 (33)	303.0	0.915 (92)	303.0	0.896 (58)
312.6	1.606 (39)	307.8	1.294 (13)	307.8	0.965 (13)	307.7	1.028 (21)
317.3	1.915 (44)	310.2	1.397 (19)	310.2	1.015 (44)	310.1	1.001 (22)
322.3	1.969 (81)	312.7	1.562 (51)	312.5	1.210 (30)	312.6	1.122 (51)
331.9	2.868 (134)	317.3	1.807 (61)	317.4	1.388 (105)	317.5	1.367 (24)
336.8	3.197 (170)	322.3	1.910 (123)	322.3	1.611 (123)	322.2	1.502 (57)
341.6	3.493 (160)	332.0	2.636 (118)	327.1	1.758 (96)	327.0	1.593 (93)
		341.5	3.111 (127)	331.9	1.659 (39)	331.8	1.142 (114)
				336.7	1.548 (192)	336.7	0.933 (41)
				341.5	1.436 (201)	341.5	0.871 (106)

## Methods

### Molecular simulation

All simulations were performed using the open-source program *ms*2^109^. Rigid, united-atom and non-polarizable force fields based on Lennard-Jones (LJ) sites and/or superimposed point dipoles or point quadrupoles were employed. The force fields for the pure substances ${\hbox {CO}}_{2}$^104^, methane^110^, ethane^110^, isobutane^111^, benzene^80^, toluene^80^ and naphthalene^112^ were parameterized using a combination of quantum chemical calculations, experimental vapor-liquid equilibrium (VLE) data and self-diffusion coefficient measurements in the case of benzene and toluene^80^ with a procedure described in Refs.^111,113,114^. For mixtures, LJ parameters for unlike interactions were optimized using combination rules^115^ as previously described in Ref.^116^.

Transport properties were sampled with equilibrium molecular dynamics and the Green–Kubo formalism^117,118^. This formalism allows for the simultaneous sampling of intra- and Maxwell–Stefan diffusion coefficients as well as shear viscosity so that it was preferred over non-equilibrium methods. The general Green–Kubo equation relates an arbitrary transport coefficient $\Xi$ to an integral of a time-correlation function by

5 $$\begin{aligned} \Xi =\frac{1}{G}\int _0^{\infty } dt~\big \langle \dot{\mathbf {A}}(\textit {t})\cdot \dot{\mathbf {A}}\text {(0)}\big \rangle . \end{aligned}$$

Therein, *G* is a transport property-specific factor, $\textbf{A}$ the related perturbation and $\dot{\mathbf {A}}$ its time derivative. The brackets <...> denote the ensemble average. The working equations for different diffusion coefficients can be found in Ref.^119^.

The thermodynamic factor can be determined using EoS or excess Gibbs energy models that were fitted to experimental VLE data. Another approach to obtain the thermodynamic factor is through molecular simulation, where chemical potential data or Kirkwood-Buff integrals (KBI) can be utilized. In this study, the thermodynamic factor was calculated based on the microscopic structure of the mixture using Kirkwood-Buff integrals (KBI) $G_{ij}$^120^

6 $$\begin{aligned} G_{ij} = 4 \pi \int _{0}^{\infty } dr \left( g_{ij}(r)-1\right) r^2, \end{aligned}$$

where $g_{ij}\,(r)$ is the radial distribution function. Equation (6) is formulated for the grand canonical ensemble, so that corrections are necessary when applying it in the canonical ensemble^121^. The truncation method proposed by Krüger et al.^122^ was employed for this purpose. Moreover, corrections of the radial distribution function based on the method by Ganguly and van der Vegt^123^ were employed. The thermodynamic factor was not extrapolated to the thermodynamic limit, as the studied simulation volumes contained a rather large number of molecules (*N* = 5000).

Cluster formation can be characterized by the excess number of solvent molecules surrounding a given solute molecule with respect to the bulk density^64^. The so-called excess coordination number is given by^63^

7 $$\begin{aligned} N_{\text {CO2}}^{\text {ex}}=\rho _{\text {CO2}}G_{ij}^{\infty }, \end{aligned}$$

where $\rho _{\text {CO2}}$ is the density of pure ${\hbox {CO}}_{2}$ and $G_{ij}^{\infty }$ denotes the KBI between carbon dioxide and solute. Further technical simulation details are given in the SI.

*Finite size corrections.* Finite-size corrections for the intra-diffusion coefficients were implemented using the approach by Yeh and Hummer^124^. The magnitude of these corrections varied depending on the density of the system. At high densities, the corrections amounted to approximately 2.4%, while at low densities they increased up to around 10.1% for the sampled infinite dilution Fick diffusion coefficient along the three isobars *p* = 9, 10 and 12 MPa. Further, two methods were investigated to account for the finite system size in the calculation of the sampled Maxwell–Stefan diffusion coefficient. The first method, proposed by Jamali et al.^125^, involves the thermodynamic factor in the denominator of the diffusion coefficient expression. This method was evaluated alongside additional simulations conducted with varying numbers of molecules (500, 800, 1200, 2500, 5000 and 6000). Four state points were tested for each mixture. Considering the strong decrease of the thermodynamic factor near the Widom line, the correction method by Jamali et al.^125^ tended to overestimate the size correction in this region. Therefore, the correction method utilizing progressively larger systems was employed in this study. Thus, the Maxwell–Stefan diffusion coefficient for all studied systems was adjusted by approximately 10% to compensate for the finite system size effects.

### Experiment

*Apparatus*. The Taylor dispersion apparatus used in this work consisted of four modules: a carrier fluid conditioning device, the ${\hbox {CO}}_{2}$ delivery system with a solute injection valve, the air bath thermostat housing the diffusion capillary and a FT-IR detector. A detailed description of the apparatus can be found in Refs.^79,126^, here only the last module is briefly described. The conventional Taylor dispersion setup, which uses a differential refractometer as a detector, is typically suitable for a wide temperature range, but is limited to the low-pressure range below 0.5 MPa. To operate at higher pressures, the introduction of restriction tubes before the refractive index detector is necessary^5^. However, this approach perturbs the diffusion process and can lead to changes of the fluid velocity and distortion of solute dispersion. In contrast, the combination of a Taylor dispersion tube with a FT-IR spectrophotometer and a back pressure regulator allows for decompression of the flow after the detector, without causing any unwanted disturbances. It should be noted that the transmission spectra of sc${\hbox {CO}}_{2}$ exhibit three wide IR transparent regions between 850–1200, 1400–2100 and 2600–3400 cm$^{-1}$, enabling the detection of vibrational modes of solute molecules appearing within these regions.

The Taylor peak was monitored at the outlet of the dispersion tube by a FT-IR spectrophotometer (Jasco FT-IR 4100) with an accuracy of 0.01 cm$^{-1}$ and a resolution of 4 cm$^{-1}$. The spectrophotometer was equipped with a high-pressure demountable flow cell (Harrick) with a wall thickness of 150 $\mu$m, featuring optical windows made of ZnSe, which allowed for a maximum working pressure of 25 MPa. One of the advantages of the FT-IR detector is its adjustability for an optimal optical path and sample volume. If desired, the optical windows can be made from sapphire, enabling even higher working pressures of up to 50 MPa, surpassing the limitation of 30 MPa in UV high-pressure flow cells^27,43,97^. One additional advantage of the FT-IR detector is its capability to analyze absorption peaks at multiple wavenumbers, which correspond to one (or several) specific types of vibrational modes of a molecule. This allows for a more comprehensive characterization of the sample. The FT-IR data were acquired and processed using Spectra Manager v.2 by Jasco (www.jascoinc.com). Response curves, showing the variation of solute concentration over time, were obtained by monitoring absorbance spectra at specific wavenumbers associated with different molecular vibration modes. The details of the selection of these working wavenumbers, the experimental protocol and the fitting procedure have been reported in Refs.^79,126^.

*Materials.* ${\hbox {CO}}_{2}$ with a certified purity of 99.998 mol% was purchased from Air Liquide in a bottle in its vapor-liquid equilibrium state, i.e., with a nominal pressure *p* = 6.4 MPa at *T* = 298 K. Benzene and toluene were purchased from Sigma-Aldrich with a purity of 99.8 mol%, whereas n-dodecane and 1,2,3,4-tetrahydronaphtalene were bought from Acros Organics with 99% and 98% purity, respectively. All solutes were used without further purification.

### Supplementary Information

Supplementary Figures.

Supplementary Information.

Supplementary Information.

## Data Availability

Excel files containing all data generated or analyzed during this study are included in the Supplementary Information.
