# Supplementary material for: Diffusion of hydrocarbons diluted in supercritical carbon dioxide
Source: Sci Rep. 2023 Sep 26;13:16107. doi: 10.1038/s41598-023-42892-7 (PMC10522683; doi:10.1038/s41598-023-42892-7)
Supplement: Supplementary file 2 — Supplementary Information. [file 41598_2023_42892_MOESM2_ESM.pdf]

# Supplementary Information to

# Diffusion of hydrocarbons diluted in supercritical carbon dioxide

**Denis Saric[1], Gabriela Guevara-Carrion[1], Yury Gaponenko[2], Valentina Shevtsova[3,4], Jadran Vrabec[1,*]**

[1]Thermodynamics, Technical University of Berlin, Ernst-Reuter-Platz 1, 10587 Berlin, Germany
[2]MRC, CP-165/62, Université libre de Bruxelles (ULB), 50, Ave. F.D. Roosevelt, B-1050 Brussels, Belgium
[3]Fluid Mechanics Group, Faculty of Engineering, Mondragon University, 20500 Mondragon, Spain
[4]IKERBASQUE, Basque Foundation for Science, Plaza Euskadi 5, 48009 Bilbao, Spain
[*]vrabec@tu-berlin.de

### Technical simulation details

Two subsequent equilibrium molecular dynamics simulations were conducted to sample the thermodynamic and transport properties of supercritical $CO_2$ mixtures. In the first step, a simulation in the isobaric-isothermal ($NpT$) ensemble was carried out at the specified temperature, pressure and composition to determine the mixture's density. Then, this density value was specified in a second simulation in the canonical ($NVT$) ensemble, to calculate the desired mixture properties.

The simulations in the $NpT$ ensemble to determine the density were equilibrated for $3 \cdot 10^4$ time steps and followed by a production run of $2 \cdot 10^6$ steps. The simulations in the $NVT$ ensemble were equilibrated for $5 \cdot 10^5$ time steps and followed by a production run of $20 \cdot 10^6$ to $24 \cdot 10^6$ steps.

A fifth-order Gear predictor-corrector scheme with an integration time step of 0.994 fs was used to solve Newton's equations of motion. The velocity scaling algorithm was employed to control the temperature. The pressure was maintained by Andersen's barostat[1] with a piston mass of $2.2 \cdot 10^9$ kg m$^{-4}$.

The cubic simulation volume with periodic boundary conditions contained 5000 molecules throughout. The cut-off radius was set to 21 Å. Lennard-Jones long range interactions were considered analytically by means of angle averaging[2] and the reaction field method with conducting boundary conditions ($\varepsilon_{RF} = 1$) was used to consider the dipolar long-range interactions beyond the cut-off radius.

Correlation functions were calculated from an average of $4 \cdot 10^5$ independent time origins and a sampling length of 40 ps throughout. This extensive length of the autocorrelation functions was chosen to avoid long-time tail corrections. The separation between the time origins was chosen such that all correlation functions achieved time independence and decayed at least to 1/e of their normalized value. Statistical uncertainties were estimated by the block averaging technique of Flyvberg and Petersen[3]. Uncertainties of the derived thermodynamic properties were estimated by the error propagation law.

## Predictive equations

**Table S1.** Average absolute relative deviation of the eleven correlations to the present simulation data along the isobars $p$ = 9, 10 and 12 MPa, as well as to the present experimental data for $CO_2$ mixtures with benzene or toluene along the isobar $p$ = 10 MPa.

| Correlation | WC | Sassiat | LT | FW | Scheibel | TC | CK | HY | HM | mRG | mSE | this work |
|---|---|---|---|---|---|---|---|---|---|---|---|---|
| Simulation data at $p$ = 9 MPa | | | | | | | | | | | | |
| Methane | 35.2 | 18.8 | 355.7 | 26.7 | 16.3 | 55.6 | 30.9 | 38.9 | 140.6 | - | - | 15.5 |
| Ethane | 30.2 | 16.2 | 261.8 | 35.2 | 15.0 | 40.5 | 28.0 | 47.0 | 88.6 | - | - | 7.9 |
| Isobutane | 21.7 | 11.0 | 141.5 | 39.2 | 10.9 | 18.2 | 18.6 | 37.5 | 42.9 | - | - | 6.5 |
| Benzene | 9.2 | 14.7 | 99.3 | 22.1 | 14.8 | 9.2 | 6.1 | 31.2 | 24.6 | 5.6 | 21.8 | 4.6 |
| Toluene | 9.6 | 11.3 | 84.9 | 25.7 | 10.7 | 8.3 | 8.5 | 30.8 | 22.1 | 8.8 | 77.0 | 6.2 |
| Naphthalene | 9.0 | 15.2 | 46.8 | 13.1 | 13.8 | 12.2 | 11.3 | 24.3 | 16.0 | 6.7 | - | 7.0 |
| Simulation data at $p$ = 10 MPa | | | | | | | | | | | | |
| Benzene | 5.0 | 14.4 | 87.1 | 24.7 | 14.5 | 4.9 | 7.7 | 21.7 | 17.1 | 5.1 | 15.3 | 4.2 |
| Toluene | 7.9 | 10.4 | 73.9 | 26.9 | 9.5 | 6.9 | 10.4 | 23.2 | 14.5 | 10.5 | 76.0 | 7.3 |
| Naphthalene | 12.2 | 22.9 | 33.6 | 9.2 | 16.2 | 14.0 | 4.8 | 3.7 | 11.3 | 12.8 | - | 10.5 |
| Simulation data at $p$ = 12 MPa | | | | | | | | | | | | |
| Benzene | 2.9 | 14.1 | 73.9 | 25.5 | 14.2 | 2.9 | 11.6 | 13.3 | 9.6 | 4.9 | 9.9 | 5.2 |
| Toluene | 6.6 | 8.3 | 63.4 | 29.1 | 7.1 | 3.1 | 15.3 | 15.5 | 7.8 | 12.6 | 72.6 | 8.6 |
| Naphthalene | 6.5 | 18.2 | 23.6 | 12.5 | 16.3 | 14.2 | 6.2 | 8.7 | 10.7 | 7.4 | - | 5.0 |
| AARD / % | 13.0 | 14.6 | 112.1 | 24.2 | 13.3 | 15.8 | 13.3 | 24.6 | 33.8 | 8.3 | 45.4 | 7.4 |
| Experimental data at $p$ = 10 MPa | | | | | | | | | | | | |
| Benzene | 29.7 | 39.5 | 41.6 | 11.8 | 39.6 | 29.5 | 92.0 | 11.4 | 52.7 | 31.3 | 88.6 | 28.9 |
| Toluene | 22.2 | 33.1 | 30.3 | 9.9 | 32.2 | 25.3 | 91.9 | 10.4 | 58.5 | 17.2 | 83.3 | 17.4 |
| AARD / % | 26.0 | 36.3 | 36.0 | 10.8 | 35.9 | 27.4 | 91.9 | 10.9 | 55.6 | 24.3 | 86.0 | 24.2 |

The abbreviations for the correlations are WC: Wilke-Chang, LT: Lai-Tan, FW: Funazukuri-Wakao, TC: Tyn-Calus, CK: Catchpole-King, HM: Hayduk-Minhas, mRG: modified Rice-Gray, mSE: modified SE.

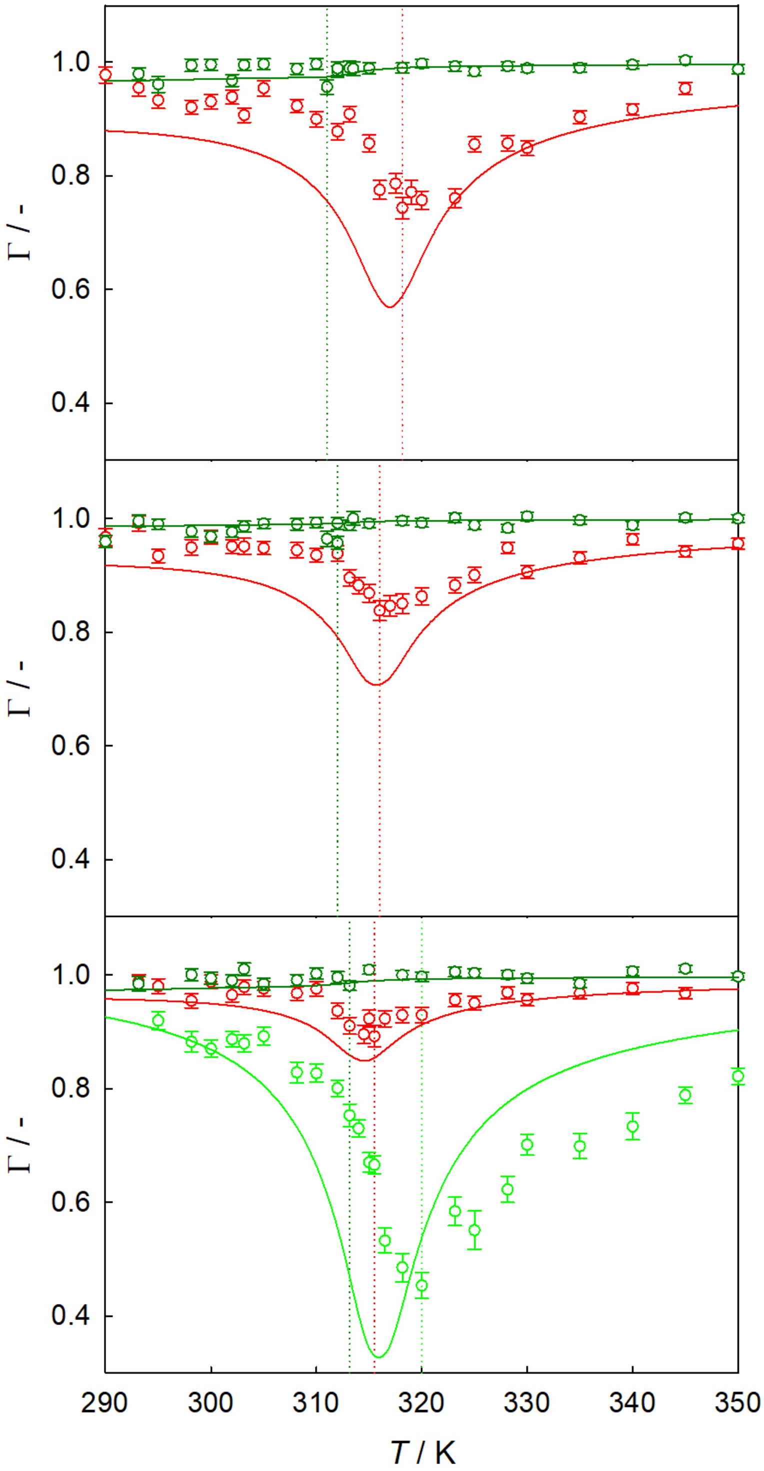


**Figure S1.** Temperature dependence of the thermodynamic factor of $CO_2$ mixtures with 0.5 mol% (bottom), 1.0 mol% (center) and 1.5 mol% (top) of ethane (dark green), benzene (red) or naphthalene (green) along the isobar $p = 9$ MPa. Circles represent molecular simulation data. Solid lines depict the thermodynamic factor calculated with TREND 5.0[4] on the basis of the GERG-2008 EoS[5] for $CO_2$ + ethane, or on the basis of the Peng-Robinson EoS for $CO_2$ + benzene ($k_{12} = 0.0967$)[6] or $CO_2$ + naphthalene ($k_{12} = 0.016, l_{12} = -0.173$)[7]. Dotted lines indicate the thermodynamic factor minimum as inferred from molecular simulation data.

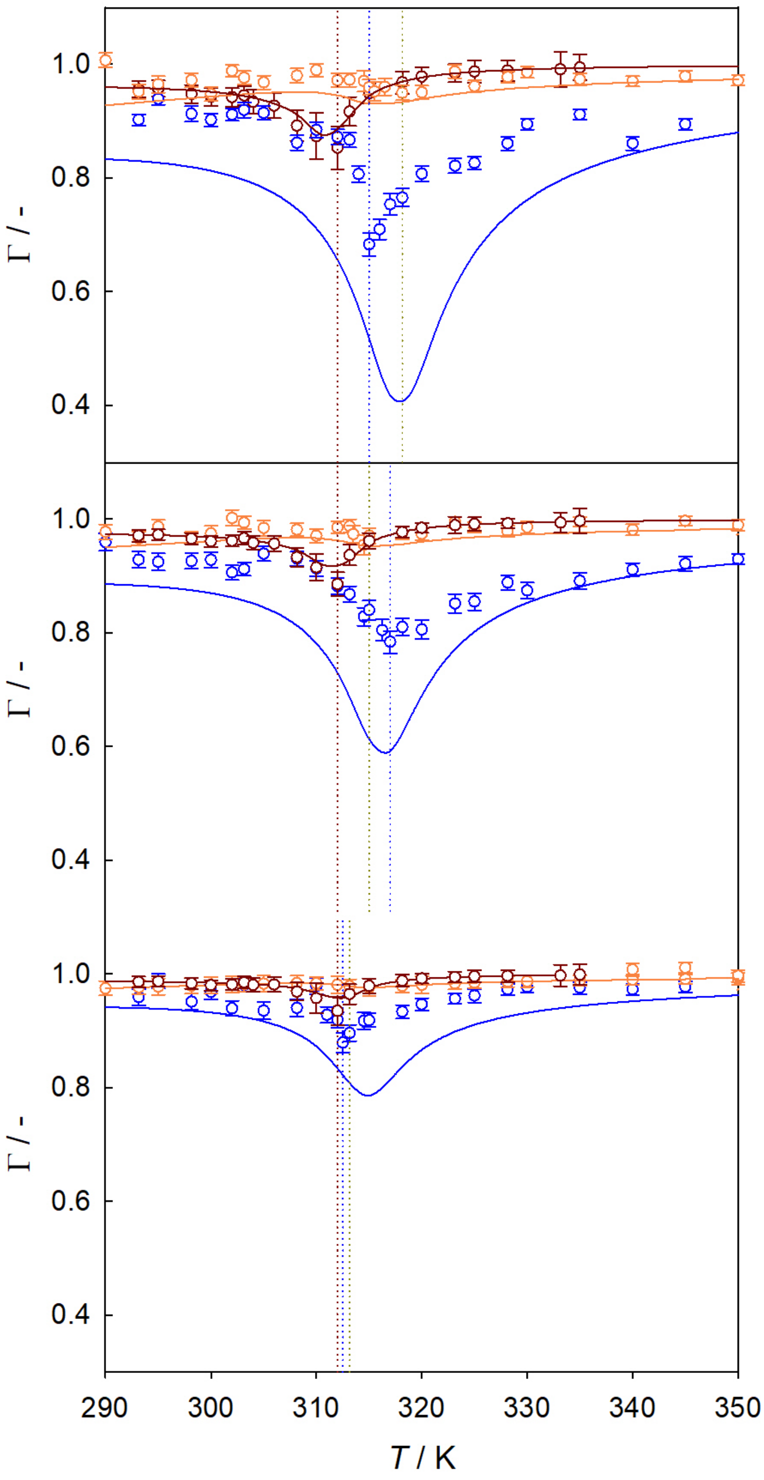


**Figure S2.** Temperature dependence of the thermodynamic factor of $CO_2$ mixtures with 0.5 mol% (bottom), 1.0 mol% (center) and 1.5 mol% (top) of methane (dark red), isobutane (orange) or toluene (blue) along the isobar $p = 9$ MPa. Circles represent molecular simulation data. Solid lines depict the thermodynamic factor calculated with TREND 5.0[4] on the basis the GERG-2008 EoS[5] for $CO_2$ + methane and $CO_2$ + isobutane, or on the basis of the Peng-Robinson EoS for $CO_2$ + toluene ($k_{12} = 0.108$)[6]. Dotted lines indicate the thermodynamic factor minimum as inferred from molecular simulation data.

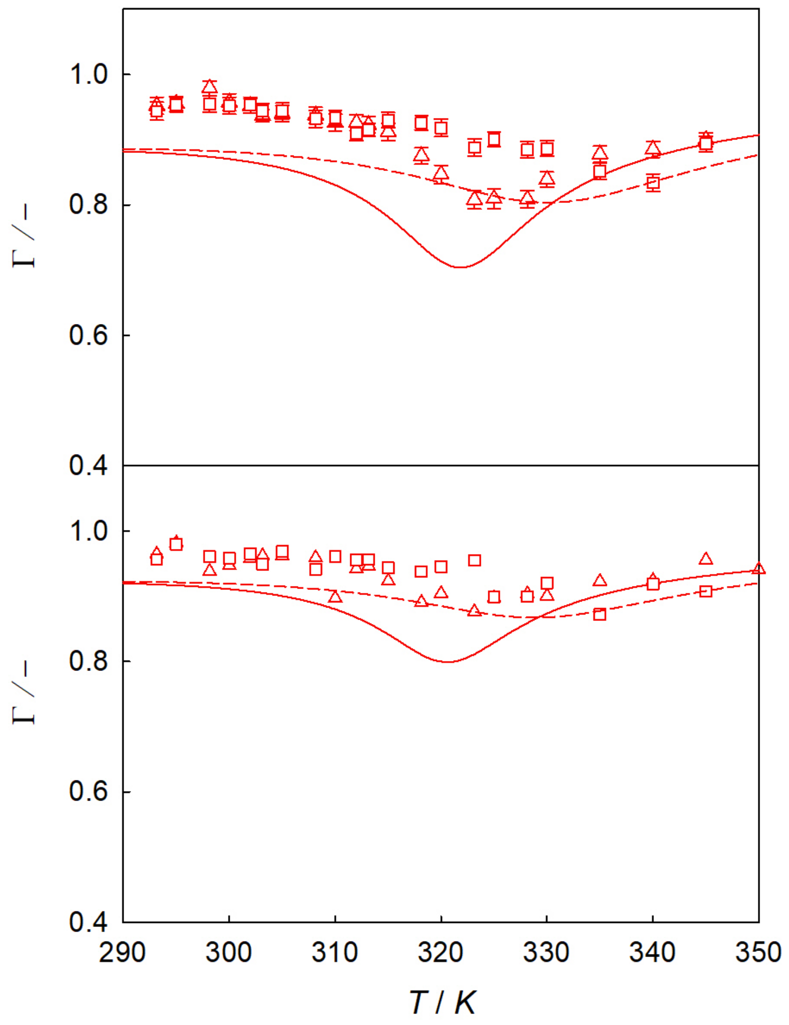


**Figure S3.** Temperature dependence of the thermodynamic factor of $CO_2$ mixtures with 1.0 mol% (bottom) and 1.5 mol% (top) of benzene along the isobars $p = 10$ MPa (triangles) and 12 MPa (squares). Symbols represent molecular simulation data at $p = 9$ MPa. Solid and dashed lines depict the thermodynamic factor calculated with TREND 5.0[4] on the basis of the Peng-Robinson EoS for $CO_2$ + benzene at 10 and 12 MPa, respectively.

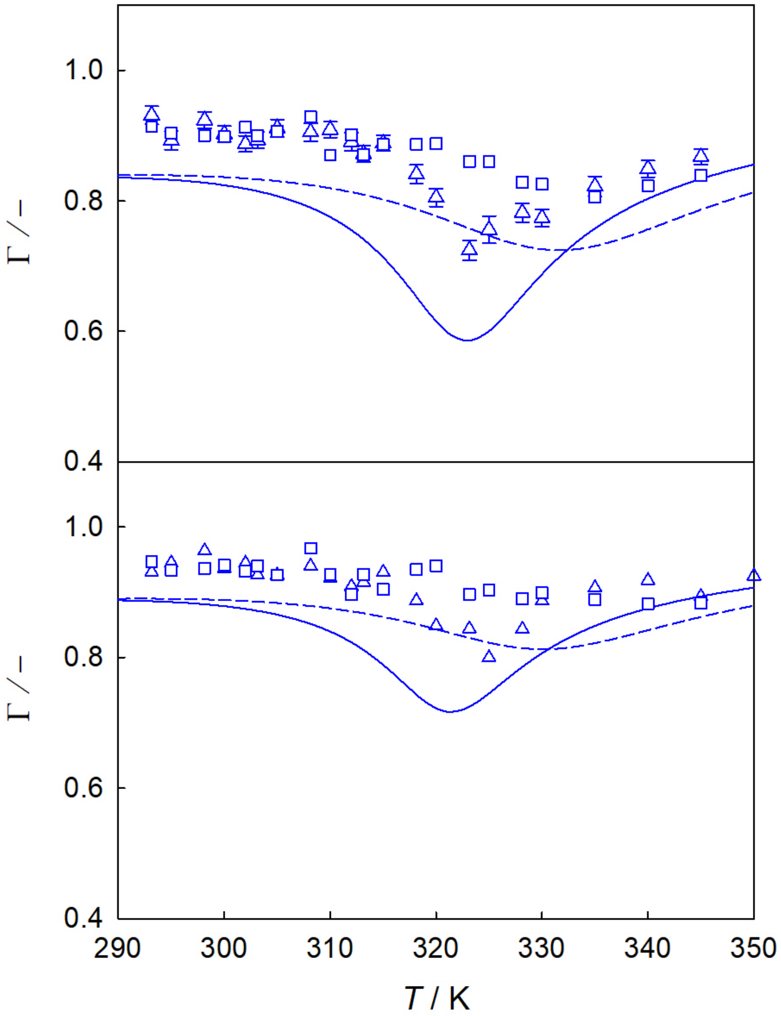


**Figure S4.** Temperature dependence of the thermodynamic factor of $CO_2$ mixtures with 1.0 mol% (bottom) and 1.5 mol% (top) of toluene along the isobars $p = 10$ MPa (triangles) and 12 MPa (squares). Symbols represent molecular simulation data at $p = 9$ MPa. Solid and dashed lines depict the thermodynamic factor calculated with TREND 5.0[4] on the basis of the Peng-Robinson EoS for $CO_2$ + toluene at 10 and 12 MPa, respectively.

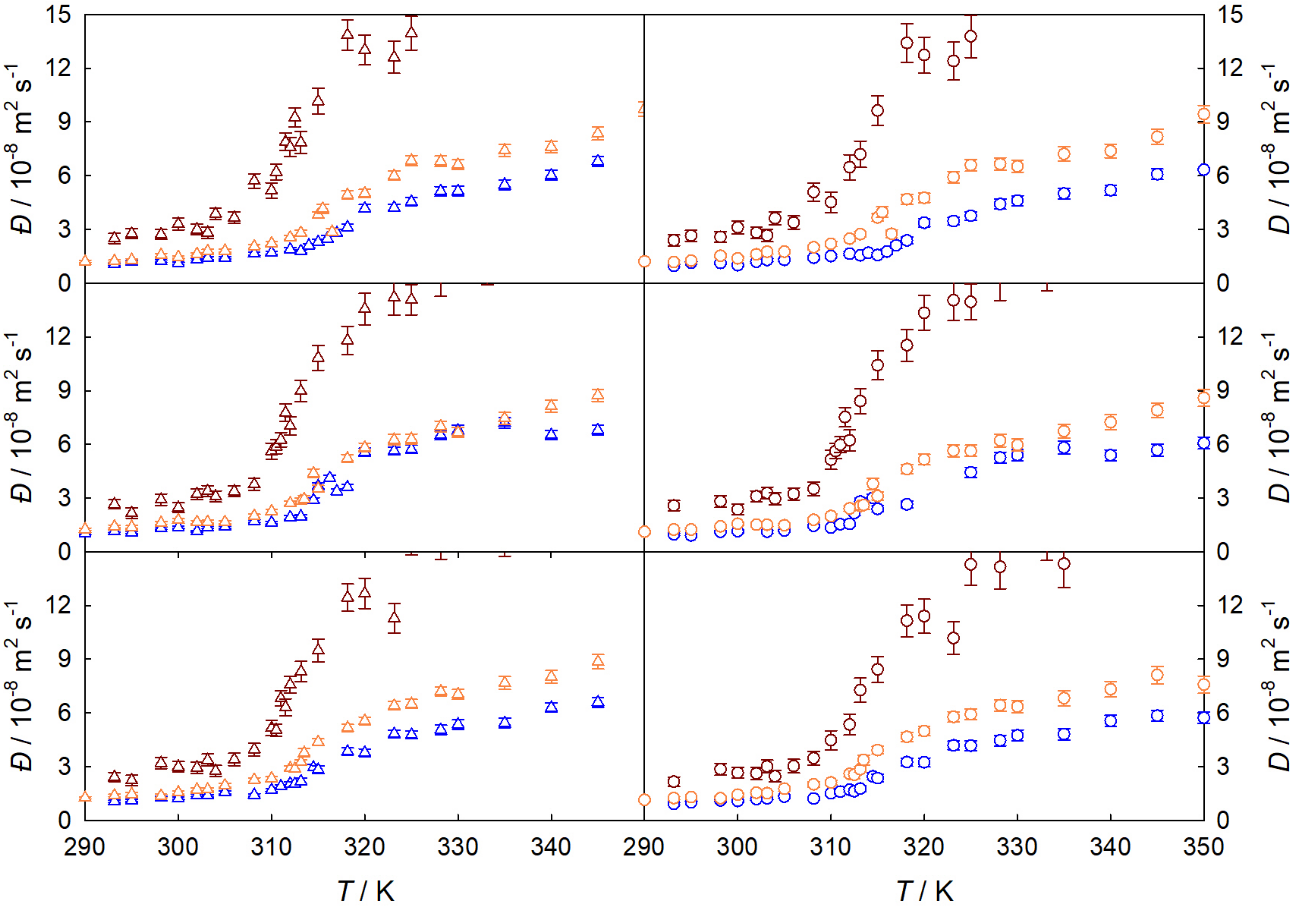


**Figure S5.** Temperature dependence of the Maxwell-Stefan diffusion coefficient (left) and the Fick diffusion coefficient (right) of $CO_2$ mixtures with 0.5 mol% (bottom), 1.0 mol% (center) and 1.5 mol% (top) of methane (dark red), isobutane (orange) or toluene (blue) along the isobar $p = 9$ MPa. Symbols represent molecular simulation data.

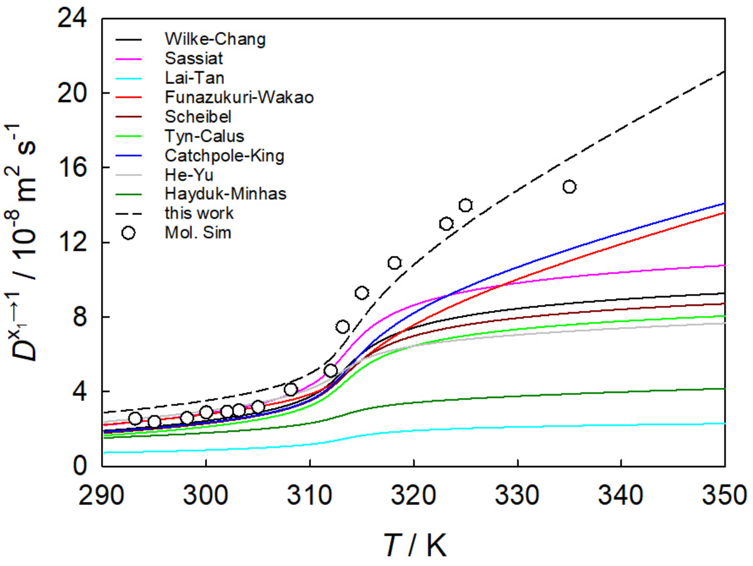


**Figure S6.** Temperature dependence of the infinite dilution Fick diffusion coefficient of $CO_2$ + methane along the isobar $p$ = 9 MPa. Circles represent molecular simulation data obtained from the extrapolation of the intra-diffusion coefficient of methane to the infinite dilution limit. Solid lines depict semi-empirical correlations [8–17] and the dashed line shows the present predictive approach (Eqs. (2) to (4)) in the paper.

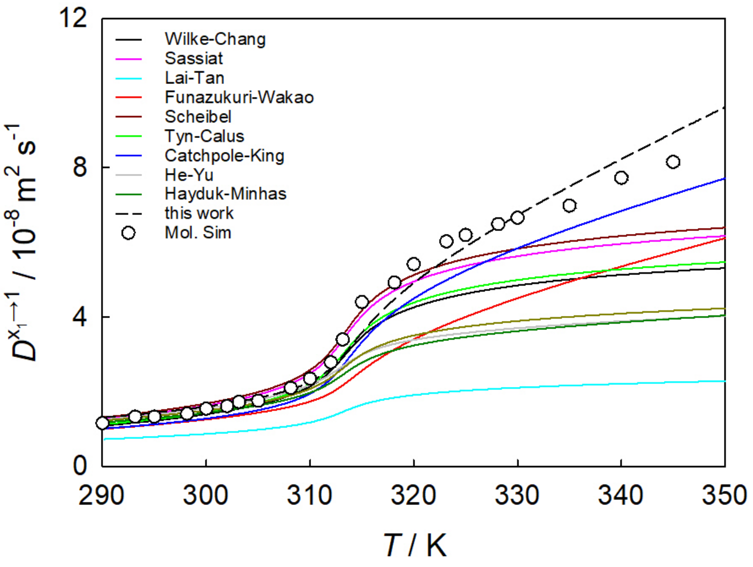


**Figure S7.** Temperature dependence of the infinite dilution Fick diffusion coefficient of $CO_2$ + isobutane along the isobar $p$ = 9 MPa. Circles represent molecular simulation data obtained from the extrapolation of the intra-diffusion coefficient of isobutane to the infinite dilution limit. Solid lines depict semi-empirical correlations [8–17] and the dashed line shows the present predictive approach (Eqs. (2) to (4)) in the paper.

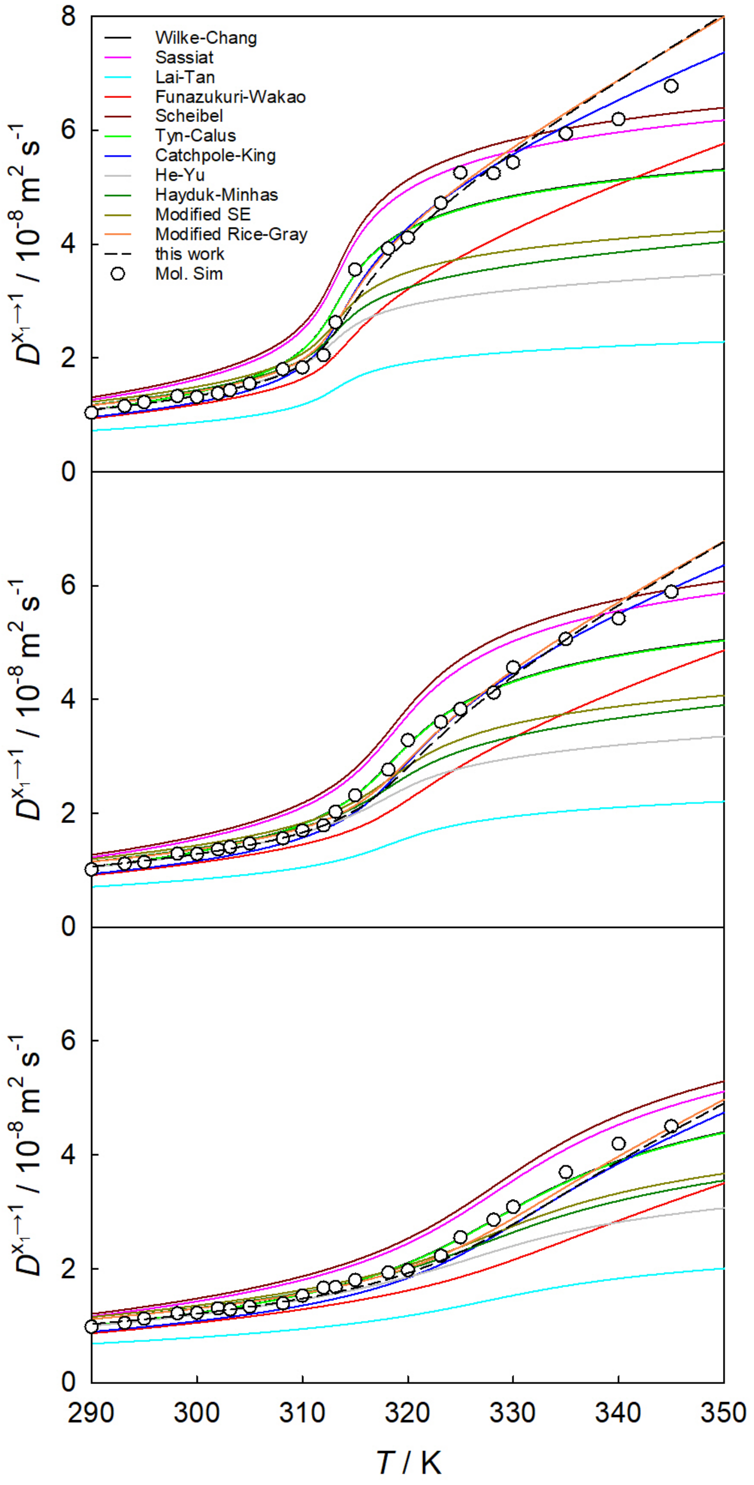


**Figure S8.** Temperature dependence of the infinite dilution Fick diffusion coefficient of $CO_2$ + benzene along the isobars $p$ = 9 MPa (top), 10 MPa (center) and 12 MPa (bottom). Circles represent molecular simulation data obtained from the extrapolation of the intra-diffusion coefficient of benzene to the infinite dilution limit. Solid lines depict semi-empirical correlations [8–17] and the dashed line shows the present predictive approach (Eqs. (2) to (4)) in the paper.

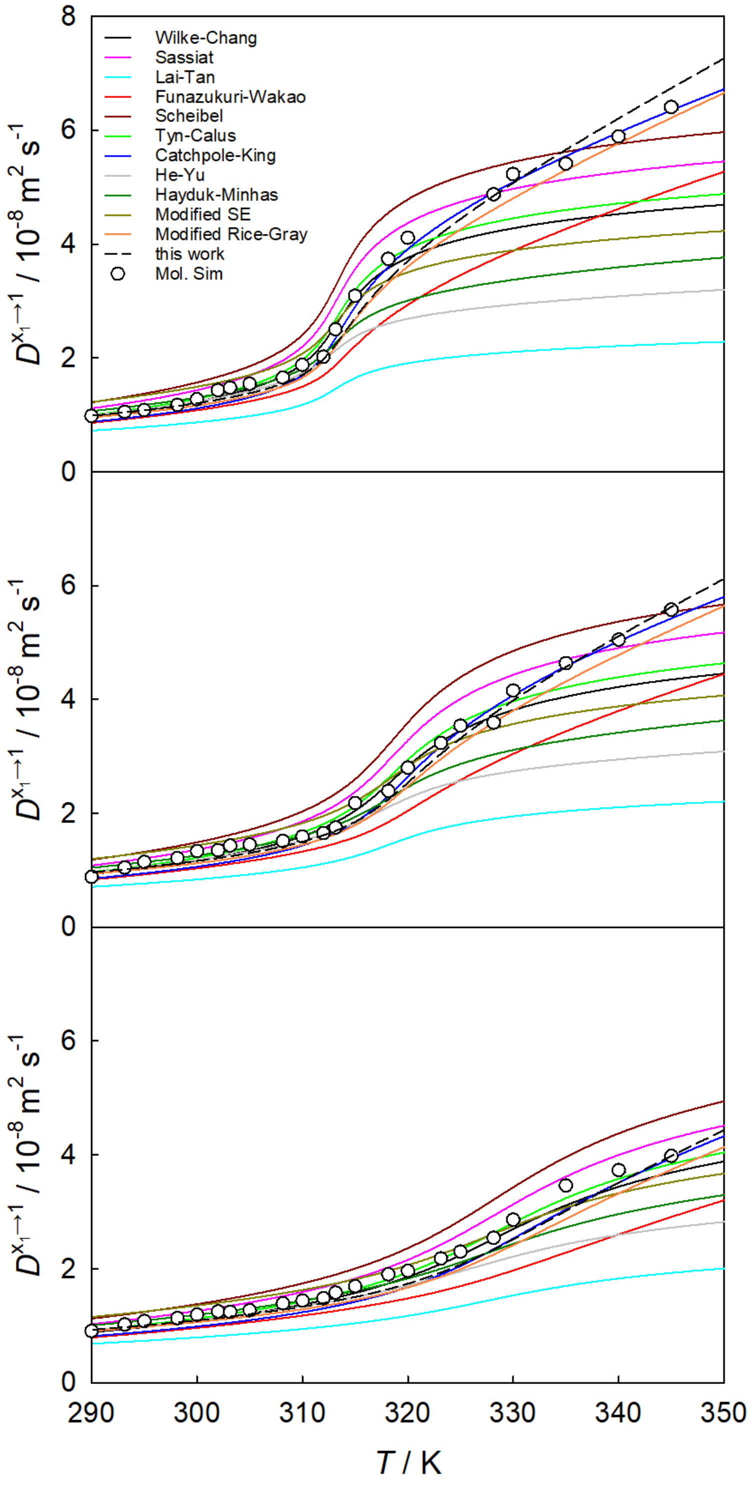


**Figure S9.** Temperature dependence of the infinite dilution Fick diffusion coefficient of $CO_2$ + toluene along the isobars $p$ = (top), 10 MPa (center) and 12 MPa (bottom). Circles represent molecular simulation data obtained from the extrapolation of the intra-diffusion coefficient of toluene to the infinite dilution limit. Solid lines depict semi-empirical correlations [8–17] and the dashed line shows the present predictive approach (Eqs. (2) to (4)) in the paper.

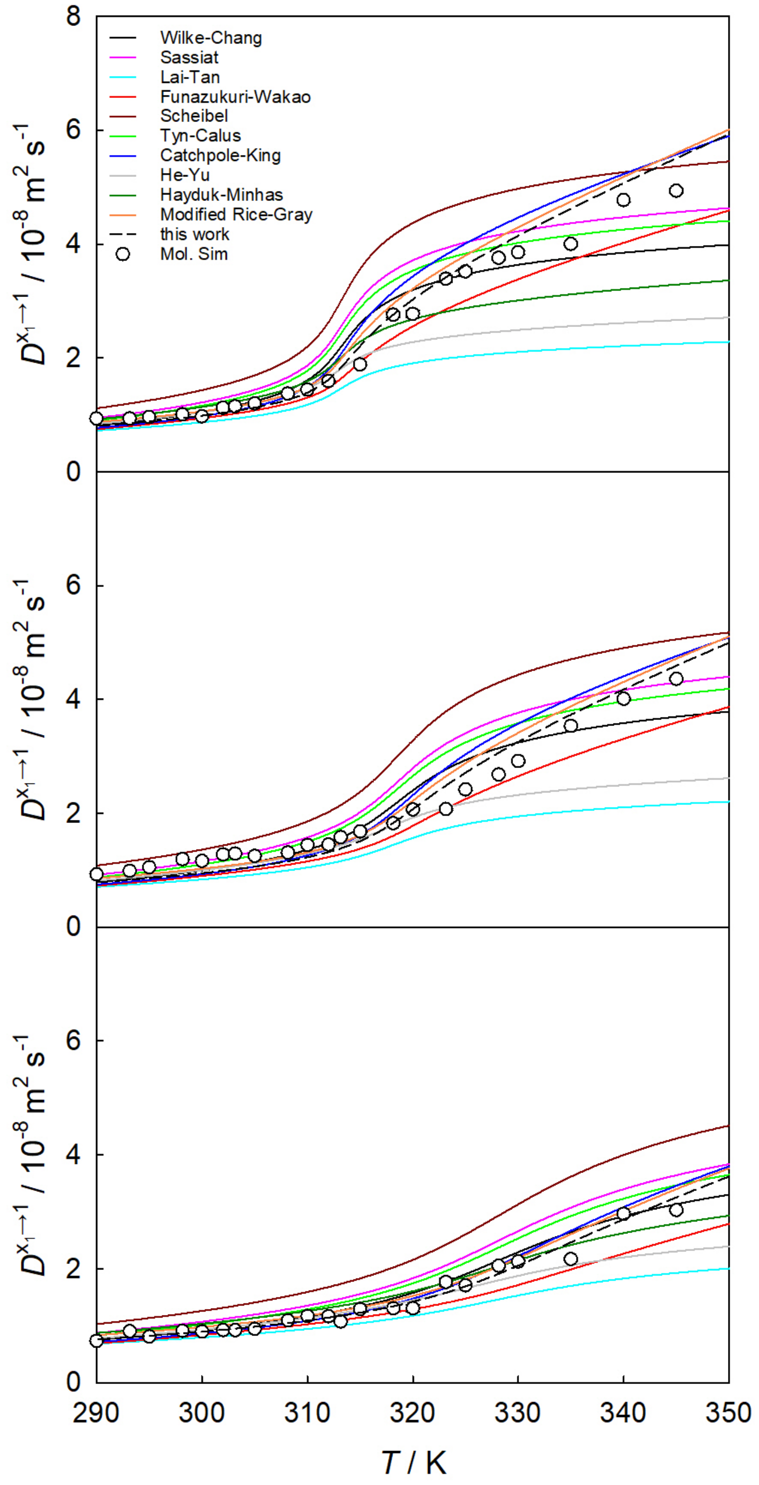


**Figure S10.** Temperature dependence of the Fick diffusion coefficient at infinite dilution of $CO_2$ + naphthalene along the isobars $p$ = 9 MPa (top), 10 MPa (center) and 12 MPa (bottom). Circles represent molecular simulation data obtained from the extrapolation of the intra-diffusion coefficient of naphthalene to the infinite dilution limit. Solid lines depict semi-empirical correlations [8–17] and the dashed line shows the present predictive approach (Eqs. (2) to (4)) in the paper.

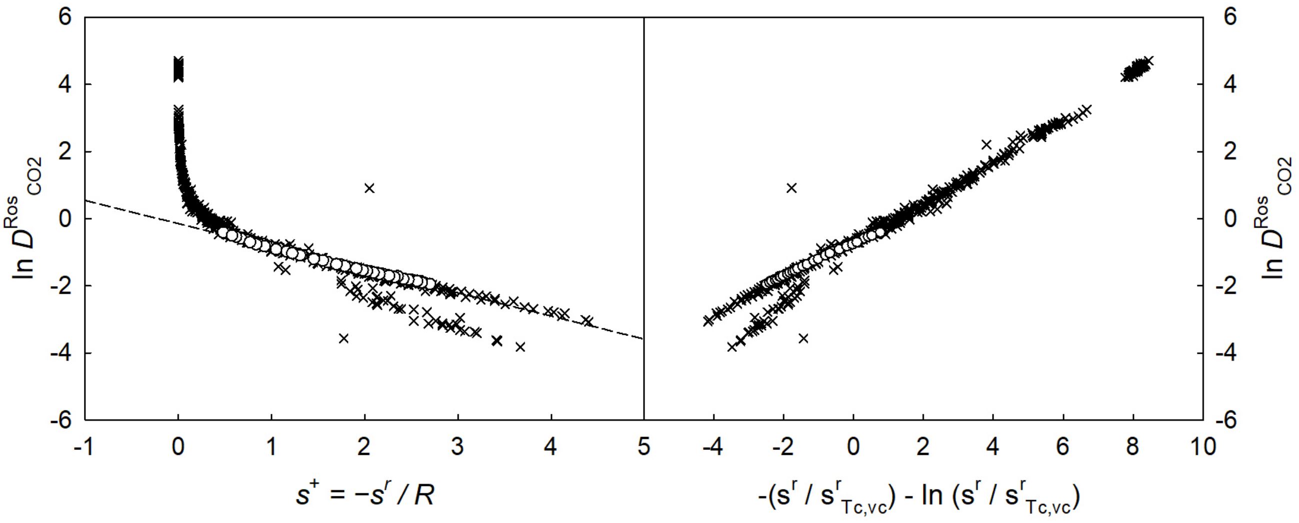


**Figure S11.** Rosenfeld's entropy scaling applied to the self-diffusion coefficient of $CO_2$ as a function of the reduced residual entropy (left) or the entropy scaling coordinate $-(s^r/s^r_{Tc,vc}) - \ln(s^r/s^r_{Tc,vc})$ [18,19] (right). Circles represent simulation data. Experimental data for the self-diffusion coefficient of $CO_2$ in combination with the reduced residual entropy obtained from the Span-Wagner EOS[20] are depicted by crosses.

## References


1. Andersen, H. C. Molecular dynamics simulations at constant pressure and/or temperature. *J. Chem. Phys.* **72**, 2384–2393 (1980).
2. Lustig, R. Angle-average for the powers of the distance between two separated vectors. *Mol. Phys.* **65**, 175–179 (1988).
3. Flyvbjerg, H. & Petersen, H. G. Error estimates on averages of correlated data. *J. Chem. Phys.* **91**, 461–466 (1989).
4. Span, R. *et al.* TREND. Thermodynamic Reference and Engineering Data 5.0 (2021). Lehrstuhl für Thermodynamik, Ruhr-Universität Bochum, Germany.
5. Kunz, O. & Wagner, W. The GERG-2008 Wide-Range Equation of State for Natural Gases and other Mixtures: An Expansion of GERG-2004. *J. Chem. Eng. Data* **57**, 3032–3091 (2012).
6. Kim, C.-H., Vimalchand, P. & Donohue, M. D. Vapor-liquid equilibria for binary mixtures of carbon dioxide with benzene, toluene and p-xylene. *Fluid Phase Equilib.* **31**, 299–311 (1986).
7. Higashi, H., Iwai, Y., Oda, T., Nakamura, Y. & Arai, Y. Concentration dependence of diffusion coefficients for supercritical carbon dioxide+ naphthalene system. *Fluid Phase Equilib.* **194**, 1161–1167 (2002).
8. Wilke, C. & Chang, P. Correlation of diffusion coefficients in dilute solutions. *AIChE J.* **1**, 264–270 (1955).
9. Scheibel, E. G. Correspondence. Liquid Diffusivities. Viscosity of Gases. *Ind. Eng. Chem.* **46**, 2007–2008 (1954).
10. Tyn, M. T. & Calus, W. F. Diffusion coefficients in dilute binary liquid mixtures. *J. Chem. Eng. Data* **20**, 106–109 (1975).
11. Hayduk, W. & Minhas, B. S. Correlations for prediction of molecular diffusivities in liquids. *Can. J. Chem. Eng.* **60**, 295–299 (1982).
12. Sassiat, P. R., Mourier, P., Caude, M. H. & Rosset, R. H. Measurement of diffusion coefficients in supercritical carbon dioxide and correlation with the equation of Wilke and Chang. *Anal. Chem.* **59**, 1164–1170 (1987).
13. Lai, C.-C. & Tan, C.-S. Measurement of molecular diffusion coefficients in supercritical carbon dioxide using a coated capillary column. *Ind. Eng. Chem. Res.* **34**, 674–680 (1995).
14. Funazukuri, T., Kong, C. Y. & Kagei, S. Binary diffusion coefficients in supercritical fluids: Recent progress in measurements and correlations for binary diffusion coefficients. *J. Supercrit. Fluids* **38**, 201–210 (2006).
15. Catchpole, O. J. & King, M. B. Measurement and correlation of binary diffusion coefficients in near critical fluids. *Ind. Eng. Chem. Res.* **33**, 1828–1837 (1994).
16. He, C.-H. & Yu, Y.-S. New equation for infinite-dilution diffusion coefficients in supercritical and high-temperature liquid solvents. *Ind. Eng. Chem. Res.* **37**, 3793–3798 (1998).
17. Funazukuri, T., Ishiwata, Y. & Wakao, N. Predictive correlation for binary diffusion coefficients in dense carbon dioxide. *AIChE J.* **38**, 1761–1768 (1992).
18. Dehlouz, A., Privat, R., Galliero, G., Bonnissel, M. & Jaubert, J.-N. Revisiting the Entropy-Scaling concept for shear-viscosity estimation from Cubic and SAFT equations of state: application to pure fluids in gas, liquid and supercritical states. *Ind. Eng. Chem. Res.* **60**, 12719–12739 (2021).
19. Dehlouz, A., Jaubert, J.-N., Galliero, G., Bonnissel, M. & Privat, R. Entropy scaling-based correlation for estimating the self-diffusion coefficients of pure fluids. *Ind. Eng. Chem. Res.* **61**, 14033–14050 (2022).
20. Span, R. & Wagner, W. A new equation of state for carbon dioxide covering the fluid region from the triple-point temperature to 1100 K at pressures up to 800 MPa. *J. Phys. Chem. Ref. Data* **25**, 1509–1596 (1996).
